# Supplementary figures and images for: Evaluation of a multimodal pain therapy approach with relapse prophylaxis for back pain (MMS-RFP study): a study protocol for a cluster randomised controlled trial
Source: BMJ Open. 2023 Jun 22;13(6):e067412. doi: 10.1136/bmjopen-2022-067412 (PMC10314416; doi:10.1136/bmjopen-2022-067412)

# Germany study region lower saxony

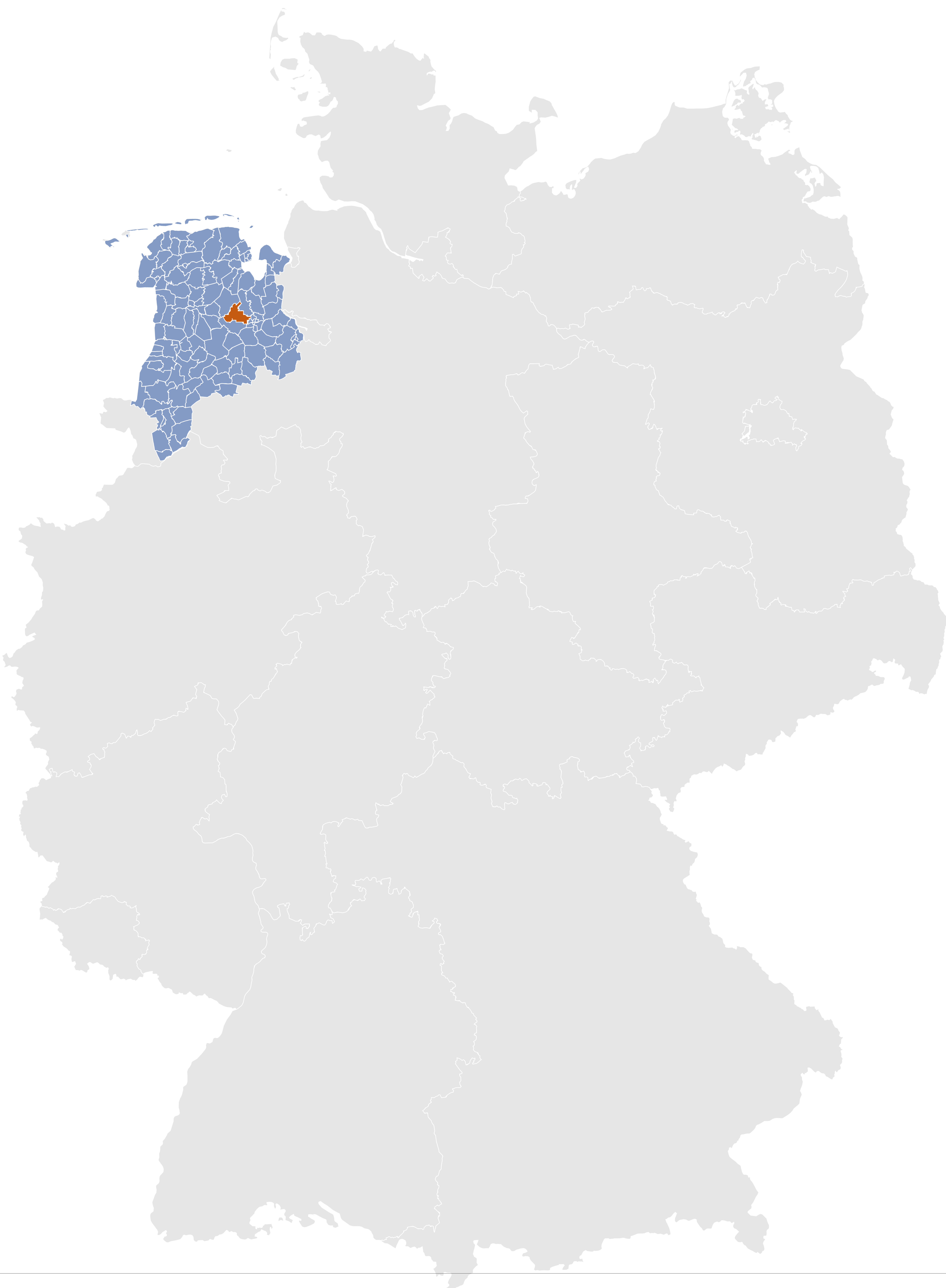

Supplement: Supplementary data [file bmjopen-2022-067412supp001.pdf]
