# Supplementary material for: Evaluation of a multimodal pain therapy approach with relapse prophylaxis for back pain (MMS-RFP study): a study protocol for a cluster randomised controlled trial
Source: BMJ Open. 2023 Jun 22;13(6):e067412. doi: 10.1136/bmjopen-2022-067412 (PMC10314416; doi:10.1136/bmjopen-2022-067412)
Supplement: Supplementary data [file bmjopen-2022-067412supp002.pdf]

t tests – Means: Difference between two independent means (two groups)

**Analysis:**	A priori: Compute required sample size		
**Input:**	Tail(s)	=	Two
	Effect size d	=	0.3409275
	α err prob	=	0.05
	Power (1–β err prob)	=	0.80
	Allocation ratio N2/N1	=	1
**Output:**	Noncentrality parameter δ	=	2.8216771
	Critical t	=	1.9687238
	Df	=	272
