# Supplementary material for: Evaluation of a multimodal pain therapy approach with relapse prophylaxis for back pain (MMS-RFP study): a study protocol for a cluster randomised controlled trial
Source: BMJ Open. 2023 Jun 22;13(6):e067412. doi: 10.1136/bmjopen-2022-067412 (PMC10314416; doi:10.1136/bmjopen-2022-067412)
Supplement: Author's manuscript [file bmjopen-2022-067412.draft_revisions.pdf]

BMJ Open

BMJ Open is committed to open peer review. As part of this commitment we make the peer review history of every article we publish publicly available.

When an article is published we post the peer reviewers' comments and the authors' responses online. We also post the versions of the paper that were used during peer review. These are the versions that the peer review comments apply to.

The versions of the paper that follow are the versions that were submitted during the peer review process. They are not the versions of record or the final published versions. They should not be cited or distributed as the published version of this manuscript.

BMJ Open is an open access journal and the full, final, typeset and author-corrected version of record of the manuscript is available on our site with no access controls, subscription charges or pay-per-view fees (http://bmjopen.bmj.com).

If you have any questions on BMJ Open's open peer review process please email info.bmjopen@bmj.com

BMJ Open

Evaluation of a multimodal pain therapy approach with relapse prophylaxis (MMS-RFP study): study protocol for a cluster randomized controlled trial

Journal:	*BMJ Open*
Manuscript ID	bmjopen-2022-067412
Article Type:	Protocol
Date Submitted by the Author:	12-Aug-2022
Complete List of Authors:	Krueger, Kathrin; Hannover Medical School, Institute for Epidemiology, Social Medicine and Health Systems Research; Center For Health Economics Research<br>Schmetsdorf, Julia; Hannover Medical School, Institute for Epidemiology, Social Medicine and Health Systems Research; Center For Health Economics Research<br>Pavlovic, Maja; AOK Niedersachsen<br>Runde, Werner; Seaside Rehabilitation Hospital Bad Zwischenahn<br>Zechel, Georg; Seaside Rehabilitation Hospital Bad Zwischenahn<br>Hemken, Norbert; Seaside Rehabilitation Hospital Bad Zwischenahn<br>Krauth, Christian; Hannover Medical School, Institute for Epidemiology, Social Medicine and Health Systems Research; Center For Health Economics Research
Keywords:	Back pain < ORTHOPAEDIC & TRAUMA SURGERY, HEALTH ECONOMICS, PAIN MANAGEMENT

SCHOLARONE™
Manuscripts

Evaluation of a multimodal pain therapy approach with relapse prophylaxis (MMS-RFP study): study protocol for a cluster randomized controlled trial

Kathrin Krueger[a,b¶], Julia Schmetsdorf[a,b¶], Maja Pavlovic[c], Werner Runde[d], Georg Zechel[d], Norbert Hemken[d], Christian Krauth[a,b]

a Institute for Epidemiology, Social Medicine, and Health Systems Research, Hannover Medical School, Hannover, Germany

b Center for Health Economics Research (CHERH), Hannover, Germany

c AOK Niedersachsen, Hannover, Germany

d Rehabilitation Center, Outpatient department for orthopaedic rehabilitation, Bad Zwischenahn, Germany

Kathrin Krüger and Julia Schmetsdorf contributed equally to this paper.

Keywords: Health economic evaluation, back pain, relapse prevention, interdisciplinary multimodal pain management, early interdisciplinary assessment

Corresponding Author:	Julia Schmetsdorf
Corresponding Author's address:	Carl-Neuberg-Str. 1
	30625 Hannover
	Germany
Corresponding Author's telephone number:	+49 511 532 4449
Corresponding Author's e-mail:	schmetsdorf.julia@mh-hannover.de

Word count: 3,960

For peer review only

For peer review only - http://bmjopen.bmj.com/site/about/guidelines.xhtml

ABSTRACT

Introduction: The need for an interdisciplinary multimodal approach to the treatment of back pain has already been demonstrated by various studies. However, when considering the periods of incapacity to work in the longitudinal course after the multimodal pain therapy (MPT), limits in terms of its sustainable effect become clear. Patients who receive MPT subsequently return to standard outpatient care, which is associated with a risk of relapse. A twelve-month relapse prophylaxis program was developed following a four-week MPT to help patients make the transition to health-conscious and physically active behaviour in everyday life and to identify and prevent impending relapses at an early stage. The evaluation seeks to provide information on the benefits of early and intensive RP as part of MPT, whether it is cost-effective, reduces the days of incapacity to work and increases functional capacity as well as to examine other parameters.

Methods and analysis: The study population comprises members of a regional statutory health insurance fund in Germany, who are ≤ 62 years old, gainfully employed and have been incapacitated for work for at least 21 days due to a diagnosis of back pain. Over a recruitment period of 24 months, a maximum of 368 individuals can potentially be included in the MPT. The evaluation is carried out on the following levels: structural, process and results quality. Cost-effectiveness is also assessed by means of a health economic evaluation. In addition to the collection of qualitative and quantitative primary data, routine data from the regional health insurance fund are also included in the analysis.

Ethics and dissemination: This study has received approval by the ethics committee of the Hannover Medical School (reference number 8548_BO_S_2019) and is registered at German Clinical Trials Register DRKS00017654. The study results will be disseminated in national and international journals and conference presentations.

Word count: 299

For peer review only - http://bmjopen.bmj.com/site/about/guidelines.xhtml

ARTICLE SUMMARY

Strengths and limitations of this study

- A comprehensive evaluation of this new treatment approach in form of relapse prevention will be provided to treat back pain adequately and furthermore support patients in their behaviour and motivation to take care of their back health themselves before chronicity occurs. At the same time, the question if people will be strengthened in their self-efficacy and self-concordance in physical activity to pursue and achieve their health goals in the long term will be examined.
- The results will contribute a better understanding if the acceptance of the programme could be increased by involving and closely accompanying the patients and providing them concrete training instructions. This highlights high demands on the treatment concept, which are not covered by standard outpatient care.
- The primary outcome is not collected via questionnaires but rather via claims data, so that an information bias can be excluded in this respect.
- As a cluster-randomized study with monthly randomization, a certain degree of contamination is unavoidable, for example, due to personnel changes, which is a limitation of the methodology. Moreover the cluster design of this trial entails some potential for selection bias.
- The nature of the relapse prophylaxis does not allow participant and therapist blinding.

For peer review only - http://bmjopen.bmj.com/site/about/guidelines.xhtml

INTRODUCTION

Low back pain has a high prevalence in today's society and is one of the most common complaints in the population. In addition to its frequent occurrence, the main criteria for the assessment of low back pain are severity, the extent of functional impairment and prognosis with regards to the return to functional and thus work capacity. Data on the prevalence of back pain in Germany include results from several regional and national surveys. The range of the prevalence lies between 30 and 70%, depending on the period considered (point prevalence, 7-day, 3-month, 1-year prevalence) [1]. The lifetime prevalence for back pain in Germany ranges from 74 to 85% [2]. In terms of chronic back pain, the lifetime prevalence lies between 24 and 30% [3]. Koes et al. stated that about 90% of all patients with low back pain met the criteria for non-specific low back pain [4]. In cases of non-specific low back pain, no clear evidence of a specific cause can be identified [4, 5]. With regards to incapacity to work, there are various references in the literature [2, 6–8]. 68-86% of patients with acute low back pain return to work within one month [6]. The recurrence rate of low back pain is 47-54%, the relapse rate into inability to work is 33% [1, 7]. A 2016 analysis of accounting data from German statutory health insurance patients concluded an increase in diagnosed lower back pain from 4.4% to 4.8% between the years 2010 and 2014. [5].

It is important to emphasize that musculoskeletal disorders are among the most expensive diseases in industrialized countries [5]. According to the 2008 Federal Health Report, the costs of non-specific low back pain amounted to 3.6 billion euros [2, 5]. The majority of these costs are due to chronic low back pain and indirect costs resulting from the loss of patients' ability to work. Based on back pain-related survey data of a large German adult sample conducted between 2003 and 2006, Wenig et al. estimated the average total costs of back pain per patient to be 1,322 € per year [9, 10].

Low back pain has been the most common reason for incapacity to work and medical rehabilitation for years. As a cause of premature retirement due to reduced earning capacity,

For peer review only - http://bmjopen.bmj.com/site/about/guidelines.xhtml

musculoskeletal disorders have taken second place in recent years after mental illness and behavioral disorders [2, 5]. A large percentage of the elderly population suffers from one or more musculoskeletal disorders. However, chronic back pain, reported by about a quarter of women and 17% of men in Germany, often affects younger people in working life. [11].

The Clinical Practice Guideline Non-Specific Low Back Pain (LBP) classifies LBP as acute (lasting up to six weeks), subacute (six to twelve weeks), or chronic (more than twelve weeks) [5]. The pain intensity may vary during these periods. Prognostically, it is difficult to determine the transition from acute to chronic pain because of the typically chronic relapsing and remitting course of back pain and its variable character [1]. Chronic recurrent refers to the recurrence of pain after a shorter or longer period without symptoms. In this regard, Pfingsten et al. suggest that outpatient treatment should be provided to patients who have a comparably short history of pain, i.e., who are in an early stage of chronification [12]. According to Brömme et al., several studies indicated that a longer duration of sick leave is associated with a lower likelihood for individuals of returning to work [13].

Back pain is usually treated in a monodisciplinary manner, although psychosocial risk factors in particular often hinder recovery and require a multimodal approach with close content-related therapy consultation [12, 14]. Interdisciplinary multimodal treatment is not yet implemented in standard outpatient care. Another problem is the relatively long period between the beginning of complaints and the presentation in a center offering multimodal treatment.

Interdisciplinary multimodal pain therapy describes the simultaneous, comprehensive treatment in which various somatic, physical and psychological interventions are integrated according to a predefined treatment plan with an identified therapeutic goal. The treatment is provided in small groups with a maximum of eight patients by a team of therapists consisting of physicians, psychologists or psychotherapists and other disciplines such as physiotherapists and others [15]. MPT is currently considered the gold standard in pain

For peer review only - http://bmjopen.bmj.com/site/about/guidelines.xhtml

management and treatment for patients with chronic or potentially chronic back pain [16]. The efficacy of interdisciplinary multimodal pain therapy (MPT) in the treatment of chronic or recurrent back pain with risk of chronification has already been proven in various studies [5, 12, 15, 17]. After successful MPT, patients usually return to the monodisciplinary treatment of their general practitioner or orthopedist. As a result, the risk of recurrence remains with the end of MPT. The goal of this new approach to the treatment of low back pain is to achieve sustainability of MPT by combining MPT with relapse prophylaxis (RP). Thus, what is learned during multimodal treatment in terms of physical activity and lifestyle can be applied to everyday life in the long term.

This approach has previously been studied by Steffens et.al, who aimed to evaluate the evidence on the effectiveness of interventions for prevention of episodes of LBP and use of sick leave due to LBP [18]. Their findings were published as a systematic review in 2016. They found evidence that exercise alone (35% risk reduction for an LBP episode and 78% risk reduction for sick leave) and exercise in combination with education (45% risk reduction for an LBP episode) helped to prevent LBP up to 1 year. They also found a reduced effect size in both conditions (exercise and education or exercise alone) in the longer term (> one year) and conclude that prevention programs aimed at long-term behaviour change in physical activity habits appear to be important.

The intervention examined in this study, the twelve-month relapse prophylaxis (RP) program, immediately follows the four-week MPT. Like the MPT, the RP is run by the Rehabilitation Center Bad Zwischenahn on an outpatient basis. The RP aims to provide prophylaxis against chronifying back pain relapses, but also to support life-integrated physical activity. RP is the all-important link between the curative MPT phase beforehand and the patient's physical activity afterwards. The patient is assigned to one of three RP paths at the end of the MPT as part of an assessment during the interdisciplinary rounds and team meetings. The paths correspond to RP programs with three different intensities (low, medium and high) for which the patient is selected based on the severity of his or her complaints (see Figure 1).

For peer review only - http://bmjopen.bmj.com/site/about/guidelines.xhtml

The low intensity level includes the opportunity for telephone consultation and the maintaining of an exercise diary as well as an obligatory telephone conversation after six weeks. After six months and again after one year, a three-hour training session takes place at the Rehabilitation Center with therapeutically supervised training, performance tests and examination of the exercise diary In addition, a psychological group session is conducted.

The medium intensity level additionally includes a training session after three months and another obligatory phone call in the fourth and eighth week after the MPT.

In the case of high-intensity RP, a weekly training session (twelve weeks of 90 minutes each) under therapeutic supervision is scheduled directly after the MPT. Sixteen and twenty weeks post MPT, obligatory telephone calls with the patient are conducted. The Follow-up training course is supplemented by another session after nine months. For patients with a particularly high need for follow-up treatment, a one-week training course is offered after twelve months.

The RP offers the advantage of facilitating the patient's transition into everyday life, accompanying and encouraging progress, as well as early recognition and prevention of relapses. The further development of care thus consists of uninterrupted, seamless and long-term care of back pain patients following multimodal pain therapy. Another goal is to motivate patients during both the MPT phase and the subsequent twelve-month RP phase under multi-professional guidance to adopt a lifelong active lifestyle. While developing good intentions seems easy, turning them into actions is a challenge. For many, the hurdles to a permanently active lifestyle in everyday life are high [19]. RP therefore serves primarily to provide professional support for the patient in the conscious, voluntary translation of goals and motives into results. For patients suffering from low back pain, this can be regarded as a secondary prophylaxis against renewed pain, functional impairment and incapacity to work. In this respect, patients at risk of recurrent back pain are on the same level as patients with other chronic widespread diseases, such as diabetes or cardiovascular diseases, for which there has long been a consensus on appropriate prevention.

For peer review only - http://bmjopen.bmj.com/site/about/guidelines.xhtml

Multimodal Pain Therapy

Employees of the AOK Lower Saxony contact the potentially eligible insured and refer them to the Rehabilitation Center. Before starting MPT, a comprehensive initial examination is performed. The MPT program intensity depends on the severity of the back pain. Patients in the standard MPT group are treated for three to four hours per day, four days a week, while patients in the complex MPT are treated for five to seven hours per day, five days a week. Two groups of eight patients each are conducted in parallel. The complex therapy group provides additional treatment and emphasizes more on psychological and medical intervention aspects, while patients in the standard group are advised to exercise on their own during these times. Overall, it is expected that patients will benefit from the structured approach of the program.

Modules of the MPT program are medical lectures and seminars such as social medicine (e.g. payment of wages in the event of illness, professional reintegration), information about the MPT concept and special features of MPT and RP, basic information in different lectures (e.g. structure and function of the spine, muscles, health-disease conception: bio-psychosocial model) and seminars addressing common questions on topics such as obesity and back pain or correlations of symptoms with stress, sports, nutrition, medications, surgery, lifting and carrying. Another module consists of medical consultations once a week alternating with and without a physiotherapist or psychologist. In addition regular medical consultations take place for support in case of abnormalities in the course of the disease and for individual treatments if needed (manual therapy, medication etc.). Moreover, psychological-pain therapy lectures and seminars are carried out. These educate patients about pain and stress, mindfulness, problematic behaviour patterns and setting realistic goals. Psychological training sessions provide practical implementations in form of relaxation techniques, stress management, progressive muscle relaxation and distraction from pain. In addition to individual psychological consultation, regular psychological counseling sessions

For peer review only - http://bmjopen.bmj.com/site/about/guidelines.xhtml

are offered. Finally, physiotherapy, occupational therapy, sports therapy and a module on motivational and volitional aspects complete the offer.

Figure 1: Treatment Algorithm.

AIMS

The objective of the study is to evaluate the benefit of early and intensive relapse prevention in the context of multimodal pain therapy. The goal is to prevent the chronification of acute nonspecific low back pain and thus reduce the high direct and indirect costs. To answer this question, outcome, process and cost-effectiveness evaluations are performed. The aim of outcome evaluation is to examine the intervention effect on patient-relevant outcomes. Process evaluation examines the extent to which the intervention was carried out and how the process is perceived and evaluated from the perspective of service providers and patients. Indirect and direct costs are considered in the health economic evaluation.

From this, the following hypotheses can be derived:

- H_1: The intervention reduces the days of incapacity to work (outcome evaluation)
- H_2: The intervention increases the functional capacity (outcome evaluation)
- H_3: The intervention is perceived positively by the participating patients (process evaluation)
- H_4: The intervention reduces direct and indirect costs (health economic evaluation)

METHODS AND ANALYSIS

Study design

In order to answer the research questions, outcome, process and health economic analyses are carried out. Different research methods are used for this purpose. The comparative population is formed by randomizing the groups of participants. The division into the respective groups (IG or CG) is carried out monthly to ensure that the patients of both groups

For peer review only - http://bmjopen.bmj.com/site/about/guidelines.xhtml

are not able to exchange information with each other. Patients who start an MPT simultaneously in the Rehabilitation Center each form a cluster. These clusters are randomized to the IG or CG (see Figure 2). The evaluator informs the Rehabilitation Center monthly via email in the third week of the MPT whether the group is an IG or CG. Up to this point, neither the patient nor the treatment staff knows which group the patient belongs to. The recruitment period is 24 months.

Figure 2: Study design.

Outcome evaluation

The results will be derived from a cluster-randomized controlled trial (C-RCT). The evaluation design incorporates an IG as well as a control group (CG) and surveys at three different points in time (see Figure 2). Both groups participate in the MPT program. While IG members additionally receive relapse prevention modules, the CG undergoes usual care. The primary outcome represents the number of days of incapacity to work within one year after the end of MPT. It is assumed that in the present sample the average number of 37 days off work per year decreases in the intervention group (IG) by 40% (or 15 days) compared to the control group (CG). One secondary outcome addresses the functional capacity. It is assumed that the functional capacity in the intervention group is significantly ($p < 0.05$) higher than in the control group. In addition, patient and provider satisfaction with the intervention will be explored, including barriers, hurdles, and potentials. Direct and indirect costs are another outcome and are compared between IG and CG.

Process evaluation

To evaluate the process quality, we examine the assignment of participants to the standard and the complex program along with the distribution of the IG to low, medium and high

For peer review only - http://bmjopen.bmj.com/site/about/guidelines.xhtml

intensity RP. In addition, we consider usage of the modules (e.g. Medical Training Therapy, Psychological Support, Phone Consultation) as well as the app utilization. To examine the quality of the intervention, patients will receive additional questions about their satisfaction with individual MPT and RP modules within the survey. In order to evaluate experiences and satisfaction with the intervention, guided interviews will be conducted. These are carried out with all physicians, psychologists and physiotherapists involved.

Health economic analysis

The health economic analysis examines the costs, cost savings and cost-effectiveness of the intervention. Therefore, intervention costs, expenditures of health care utilization, indirect costs due to incapacity to work, and the patient's health-related quality of life are assessed. The evaluation is carried out based on routine data. Incremental cost-effectiveness is analysed by considering direct and indirect costs as well as the health-related quality of life of both groups, IG and CG.

Measurements

Various instruments are used in the course of primary data collection including standardised questionnaires (Table 1). Prior to the start of MPT, intervention and control group receive a questionnaire to assess the status quo at the beginning of the intervention (T0). The questionnaire comprises various items on socio-demographic and socio-economic parameters together with health-related behaviour (exercise, diet, smoking etc.). In addition, standardized survey instruments are included to assess functional capacity [20] and health-related quality of life (EQ-5D) [21]. A visual analog scale is used to assess the patient's health-related quality of life from 0-100. IG and CG receive further questionnaires after completion of the four-week MPT (T1) and twelve months post-intervention (T2). All questionnaires except for T0 include questions regarding patient satisfaction and experience with the intervention. Two weeks after handing out or sending the questionnaire, patients

For peer review only - http://bmjopen.bmj.com/site/about/guidelines.xhtml

who have not yet responded will receive a reminder to increase the response rate. A second reminder is sent after an additional ten days.

Secondary data are included in the analyses based on routine data of the regional health insurance fund AOK Lower Saxony. These include inpatient treatment, rehabilitation services, pharmaceuticals and remedies along with days of incapacity to work. Moreover, sociodemographic factors of influence (e.g. age, gender, level of education) together with data regarding health-related quality of life are included in the analysis.

Table 1. Quantitative and qualitative data sources

Target group	Data source	Data type	Instrument	Parameter
**Patients**	Primary data	Quantitative	Questionnaire	Age, gender, sociodemography, satisfaction, functional capacity, health-related quality of life, general self-efficacy, impairment due to pain, health behaviour, sports activity, nutrition, smoking, alcohol
**Medical Staff, Psychologists**	Primary data	Qualitative	Interview	Experience and satisfaction with the intervention
**Patients**	Secondary data	Quantitative	Routine data	Days of incapacity to work, region, pharmaceuticals, therapeutic products, outpatient and inpatient

For peer review only - http://bmjopen.bmj.com/site/about/guidelines.xhtml

				care

Study population

The study population comprises members of the regional health insurance fund AOK Lower Saxony, residing in the northwest of Lower Saxony (see Figure 3 and 4), who are entitled to receive sick pay and have been incapacitated for work for at least 21 days during the last six months due to a diagnosis of back pain (ICD M40-M54, excluding M45, M46 and M49). Patients with main diagnosis "mental and behavioral disorders" (ICD F00-F99; six months before), "malignant neoplasms" (ICD C00-C97; twelve months before incapacity to work) and "injuries" (ICD S00-S99; six months before incapacity to work), the diagnoses "paraparesis and paraplegia, tetraparesis and tetraplegia" (ICD G82) or an age ≥ 62 years will be excluded from the sample. Patients with one of the above-mentioned inclusion diagnoses are also excluded if the illness is already classified as chronic, i.e. at the time of the intervention, the duration of the incapacity to work within the last six months is cumulatively longer than twelve weeks.

Figure 3 and 4: Study region.

Participant recruitment

Each month, with the exception of December, two MPT courses with eight participants each can be held at the Rehabilitation Center Bad Zwischenahn. In December, a single course will be held. Over a recruitment period of 24 months, a maximum of 368 individuals could begin a MPT. The risk factors of a possibly low willingness to participate or a high dropout rate of patients was counteracted in advance by the close involvement of the specialised employees of the AOK. For example the inclusion and exclusion criteria were developed based on their

For peer review only - http://bmjopen.bmj.com/site/about/guidelines.xhtml

experience. Nevertheless, the development of the case number and the dropout rate will be subject to constant monitoring so that actions can be taken and incentives can be implemented if necessary.

Sample Size calculation

The case number calculation is carried out for the primary outcome days of incapacity to work. Currently, the described patient group of AOK-insured persons has an average number of 37 days off work (SD: 58) per year. It is assumed that the average number of 37 days off work per year decreases in the intervention group (IG) by 40% (or 15 days) compared to the control group. (CG).This results in an effect size of d = 0.3409275. To test with a power of 80% (1 - ß = 0.8) and a significance level of 5% (α error = 0.05), a case number of 137 subjects per group is required for significant proof of the expected effect. The number of cases was calculated using the software G*Power (see Figure 5).

Taking an intraclass correlation coefficient (ICC) of 0.01 and a design effect of 1.1 at an average cluster size of 15.3 (22*16+2*8) into account, the required number of cases is 293 subjects, i.e. 147 subjects per group [22–25]. Considering a drop-out of 10% for patients who drop out during the MPT, i.e. before randomization, results in a required total case number of 322. The available population covers the required case number (see Figure 6).

Figure 5: Sample size calculation.

Figure 6: CONSORT Flow Diagram.

Statistical analysis

Qualitative analysis

For peer review only - http://bmjopen.bmj.com/site/about/guidelines.xhtml

The qualitative guided interviews are documented by means of audio recordings. The recordings are transcribed without personal information (e.g. name, place of residence). The evaluation of the guided interviews is based on the qualitative content analysis according to Mayring and is carried out with the help of the MAXQDA software [26].

Quantitative analysis

All patient-related data will be pseudonymised by assigning a unique identification number. A plausibility check of the data will be carried out. Initially, descriptive analysis of the data is performed. Sample characteristics are analysed with regard to their distribution in the two groups by means of the Chi-square test for binary variables and Mann-Whitney-U-test for continuous variables.

Primary Outcome

The data will be analysed by reference to the primary outcome (days of incapacity to work), which is carried out by intention-to-treat analysis. Patients are analysed according to their original group assignment.

To test the primary hypothesis H_1 "The intervention reduces the number of days of incapacity to work", an analysis of covariances (ANCOVA) with cluster adjustment is performed, using a significance level of 0.05. Covariables included into the model are age, gender and comorbidities (e.g. depression), the prescription of painkillers, indication-related hospitalization and surgical interventions. In addition, a hypothesis test (two-sided t-test with a significance level of 0.05) is performed.

Secondary outcomes

Data for secondary outcome variables are obtained via questionnaires and routine data. The Markov Chain Monte Carlo approach (MCMC) in SPSS is used as imputation method to replace missing values [27–29]. To test the hypothesis H_2 "The intervention increases functional capacity" and H_4 "The intervention reduces direct and indirect costs", hypothesis

For peer review only - http://bmjopen.bmj.com/site/about/guidelines.xhtml

tests (one-sided t-test with a significance level of 0.05) are performed. The third hypothesis H_3 "The intervention is perceived positively by the participating patients" will be tested by conducting a pencil-paper questionnaire with the patients. The analyses are carried out using the software program SPSS.

Patient and Public Involvement

No patients were involved in the design and conduct of the study, choice of outcome measures and recruitment to the study. The results of the study will be provided to patients at their request.

ETHICS AND DISSEMINATION

Ethical and scientific standards in the currently valid version are taken into account when conducting the study, especially the Memorandum on Good Scientific Practice of the German Research Foundation [30]. In particular, analysis of secondary data is performed according to the Good Practice of Secondary Data Analysis guidelines, whereas the primary data collection is conducted in accordance with the STROBE (Strengthening the Reporting of Observational Studies in Epidemiology) statement [31]. In addition, the methods of health economic evaluation in health services research and the Hannover Consensus are applied [32]. Preliminary to the intervention, a concrete analysis plan is defined. Moreover, the questionnaires developed for the MPT and RP will be pre-tested and all data will be subjected to a validation process. In addition, participation in the intervention is voluntary for the insured, so there is no disadvantage if people do not want to participate. This study has received approval by the ethics committee of the Hannover Medical School (reference number 8548_BO_S_2019) and is registered at German Clinical Trials Register DRKS00017654. Each participating patient has signed a contract for participation in the program, and we will obtain written informed consent. Furthermore, all study participants will be comprehensively informed about the use of the collected data and the purpose and procedure of the project. All transmitted data is pseudonymised and is only accessible to

For peer review only - http://bmjopen.bmj.com/site/about/guidelines.xhtml

project members. The delivered data is stored on a secure and encrypted network drive. Access to the files is restricted to the project staff. No data will be passed on to third parties. Furthermore, the information obtained is used exclusively for research purposes and no information is passed that enables conclusions about individual persons. All data collected and processed will be stored on a secure and encrypted hard disk after the end of the study and deleted ten years after (by 31 March 2033 at the latest).

The study results will be disseminated in national and international journals and conference presentations. The Innovation Fund (G-BA) will publish the study outcomes in a results report. Additionally, the health insurance fund AOK Lower Saxony will make the results available via its homepage and member journals.

For peer review only

Author contributions

KK and CK conceived, designed and obtained funding for the research. The first draft of this manuscript was produced by KK and JS. All authors have provided input to, reviewed, edited and approved the final version. Conceptualization: KK, CK, WR, GZ. Funding acquisition: CK, MP, NH. Methodology: KK, CK. Project administration: MP. Supervision: CK. Visualization: JS, KK. Writing - original draft: JS, KK. Writing - review & editing: JS, KK, CK, MP, WR, GZ, NH.

For peer review only

Funding statement

This project is funded by the Federal Joint Committee (G-BA), Innovation Fund, (funding code 01NVF18011). The funding company had no role in study design, data collection and analysis, decision to publish, or preparation of the manuscript.

Competing interests

The authors declare that they have no competing interest.

Abbreviations

AOK	Allgemeine Ortskrankenkasse (Regional Statutory Health Insurance Fund)
CG	Control group
IG	Intervention group
RP	Relapse prevention
LBP	Low Back Pain
MPT	Multimodal pain therapy

Appendix

Not available.

Acknowledgements

For peer review only - http://bmjopen.bmj.com/site/about/guidelines.xhtml

Not applicable.

For peer review only

For peer review only - http://bmjopen.bmj.com/site/about/guidelines.xhtml

REFERENCES

1 Casser H-R, Seddigh S, Rauschmann M. Acute Lumbar Back Pain. *Dtsch Arztebl Int* 2016;113(13):223–34.

2 Raspe H-H, ed. Rückenschmerzen. Berlin: Robert-Koch-Institut (RKI) 2012.

3 Neuhauser H, Ellert U, Ziese T. Chronische Rückenschmerzen in der Allgemeinbevölkerung in Deutschland 2002/2003: Prävalenz und besonders betroffene Bevölkerungsgruppen. *Gesundheitswesen* 2005;67(10):685–93.

4 Koes BW, van Tulder MW, Thomas S. Diagnosis and treatment of low back pain. *BMJ* 2006;332(7555):1430–34.

5 Bundesärztekammer (BÄK), Kassenärztliche Bundesvereinigung (KBV), Arbeitsgemeinschaft der Wissenschaftlichen Medizinischen Fachgesellschaften (AWMF). Nationale VersorgungsLeitlinie Nicht-spezifischer Kreuzschmerz – Langfassung, 2. Auflage. Version 1 2017.

6 Pengel LHM, Herbert RD, Maher CG, et al. Acute low back pain: systematic review of its prognosis. *BMJ* 2003;327(7410):323.

7 Mehling WE, Gopisetty V, Bartmess E, et al. The prognosis of acute low back pain in primary care in the United States: a 2-year prospective cohort study. *Spine (Phila Pa 1976)* 2012;37(8):678–84.

8 Hestbaek L, Leboeuf-Yde C, Manniche C. Low back pain: what is the long-term course? A review of studies of general patient populations. *Eur Spine J* 2003;12(2):149–65. doi:10.1007/s00586-002-0508-5 [published Online First: 28 January 2003].

9 Wenig CM, Schmidt CO, Kohlmann T, et al. Costs of back pain in Germany. *Eur J Pain* 2009;13(3):280–86. doi:10.1016/j.ejpain.2008.04.005 [published Online First: 3 June 2008].

10 Schmidt CO, Raspe H, Pfingsten M, et al. Back pain in the German adult population: prevalence, severity, and sociodemographic correlates in a multiregional survey. *Spine (Phila Pa 1976)* 2007;32(18):2005–11.

11 Robert Koch-Institut. Gesundheit in Deutschland: RKI-Bib1 (Robert Koch-Institut) 2016.

12 Pfingsten M, Arnold B, Böger A, et al. Sektorenübergreifende interdisziplinäre multimodale Schmerztherapie Empfehlungen zu Struktur- und Prozessparametern der Ad-hoc-Kommission „Interdisziplinäre Multimodale Schmerztherapie“ der Deutschen Schmerzgesellschaft e. V. *Schmerz* 2019;33(3):191–203.

13 Brömme J, Mohokum M, Disch AC, et al. Interdisziplinäre, multimodale Schmerztherapie vs. konventionelle Therapie Eine Kostenanalyse bei Patienten mit chronischen Rückenschmerzen. *Schmerz* 2015;29(2):195–202.

14 Pfingsten M. Chronischer Rückenschmerz--Interdisziplinäre Diagnostik und Therapie. *Anästhesiol Intensivmed Notfallmed Schmerzther* 2009;44(1):40-5; quiz 46. doi:10.1055/s-0028-1128185 [published Online First: 29 December 2008].

15 Arnold B, Brinkschmidt T, Casser H-R, et al. Multimodale Schmerztherapie: Konzepte und Indikation. *Schmerz* 2009;23(2):112–20.

For peer review only - http://bmjopen.bmj.com/site/about/guidelines.xhtml

16 Behrendt S, Kulas H, Marschall U, et al. Which Back Pain Patients Benefit From Multidisciplinary Pain Management? A Comparative Cost Analysis Using Claims Data of Statutory Health Insurance. *Gesundheitswesen* 2016;78(S 01):e120-e127. doi:10.1055/s-0042-110401 [published Online First: 21 July 2016].

17 Kamper SJ, Apeldoorn AT, Chiarotto A, et al. Multidisciplinary biopsychosocial rehabilitation for chronic low back pain: Cochrane systematic review and meta-analysis. *BMJ* 2015;350:h444. doi:10.1136/bmj.h444 [published Online First: 18 February 2015].

18 Steffens D, Maher CG, Pereira LSM, et al. Prevention of Low Back Pain: A Systematic Review and Meta-analysis. *JAMA Intern Med* 2016;176(2):199–208.

19 Fuchs R, Goehner W, Seelig H. Long-term effects of a psychological group intervention on physical exercise and health: the MoVo concept. *J Phys Act Health* 2011;8(6):794–803.

20 Kohlmann T, Raspe H. Der Funktionsfragebogen Hannover zur alltagsnahen Diagnostik der Funktionsbeeinträchtigung durch Rückenschmerzen (FFbH-R). *Rehabilitation* 1996;35(1):I–VIII.

21 J.-Matthias Graf v. d. Schulenburg, C. Claes, W. Greiner, A. Uber. Die deutsche Version des EuroQol-Fragebogens. *Zeitschrift für Gesundheitswissenschaften* 1998;6(1):3–20.

22 Eldridge SM, Ashby D, Kerry S. Sample size for cluster randomized trials: effect of coefficient of variation of cluster size and analysis method. *Int J Epidemiol* 2006;35(5):1292–300. doi:10.1093/ije/dyl129 [published Online First: 30 August 2006].

23 Dreyhaupt J, Mayer B, Kaluscha R, et al. Cluster-randomisierte Studien: Methodische und praktische Aspekte. *Rehabilitation* 2020;59(1):54–61. doi:10.1055/a-0801-5697 [published Online First: 23 January 2019].

24 Rutterford C, Copas A, Eldridge S. Methods for sample size determination in cluster randomized trials. *Int J Epidemiol* 2015;44(3):1051–67. doi:10.1093/ije/dyv113 [published Online First: 13 July 2015].

25 Lorenz E, Köpke S, Pfaff H, et al. Cluster-Randomized Studies. *Dtsch Arztebl Int* 2018;115(10):163–68.

26 Mayring P. Qualitative Inhaltsanalyse: Grundlagen und Techniken, 11th edn. Weinheim: Beltz 2010.

27 Li K-H. Imputation using markov chains. *Journal of Statistical Computation and Simulation* 1988;30(1):57–79.

28 Rubin BD. Multiple imputation for survey nonresponse 1987.

29 Mayer B. Fehlende Werte in klinischen Verlaufsstudien - der Umgang mit Studienabbrechern 2011.

30 Vorschläge zur Sicherung guter wissenschaftlicher Praxis: Empfehlungen der Kommission "Selbstkontrolle in der Wissenschaft" ; Denkschrift. Weinheim: Wiley-VCH 2013.

31 Graf von der Schulenburg J-M, Greiner W, Jost F, et al. German recommendations on health economic evaluation: third and updated version of the Hanover Consensus.

For peer review only

For peer review only - http://bmjopen.bmj.com/site/about/guidelines.xhtml

Value Health 2008;11(4):539–44. doi:10.1111/j.1524-4733.2007.00301.x [published Online First: 11 January 2008].

32 Workgroup GPGI. Gute Praxis Gesundheitsinformation. *Z Evid Fortbild Qual Gesundhwes* 2016;110-111:85–92. doi:10.1016/j.zefq.2015.11.005 [published Online First: 13 January 2016].

For peer review only

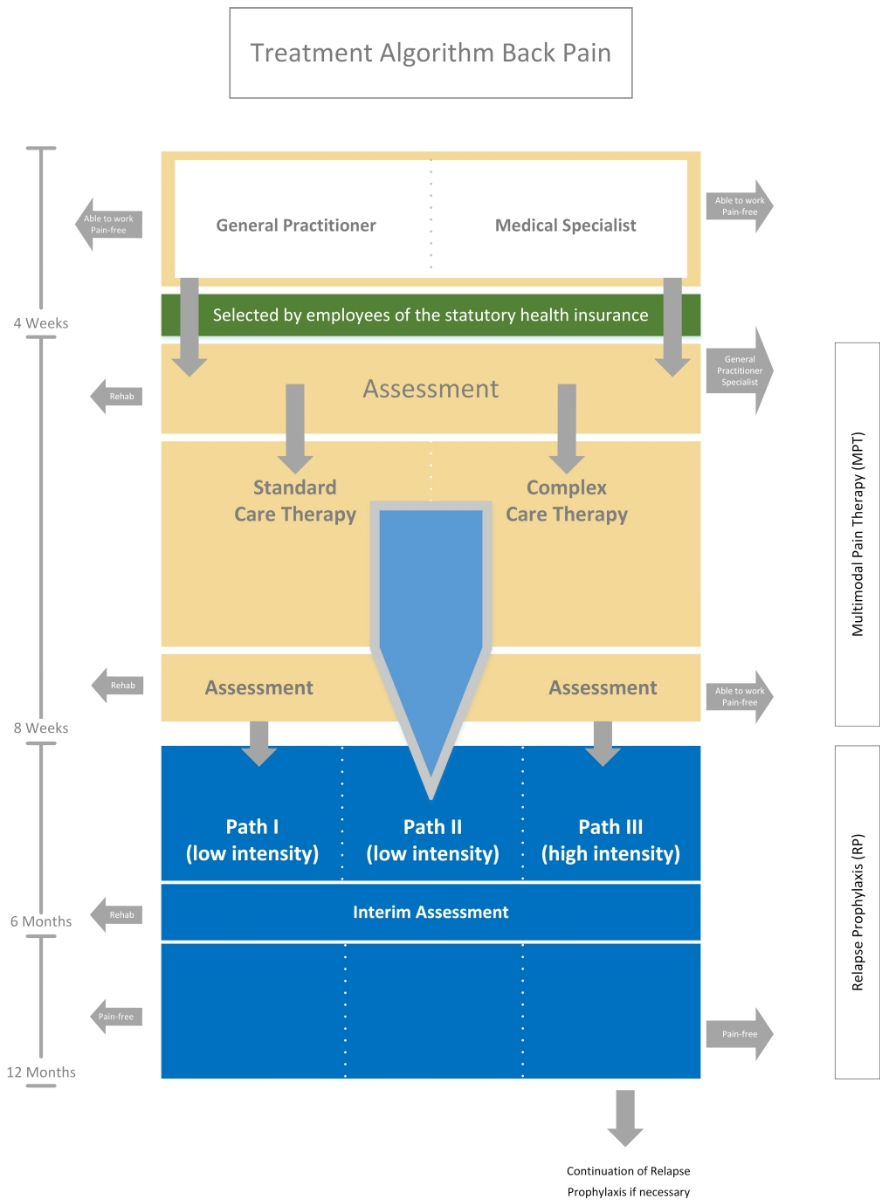

Treatment Algorithm

415x565mm (236 x 236 DPI)

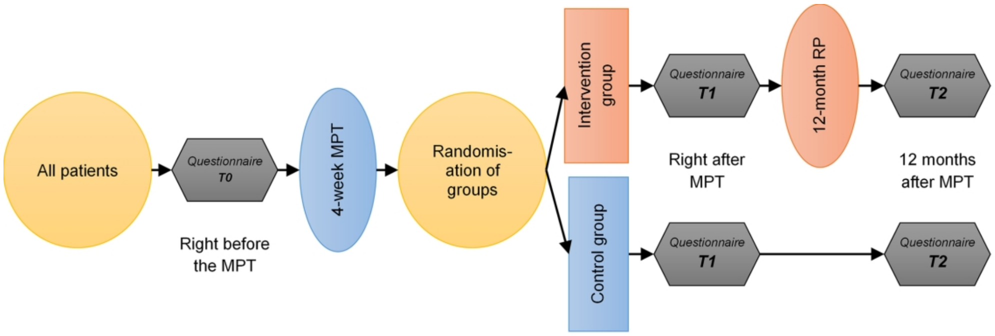

Study design

190x66mm (300 x 300 DPI)

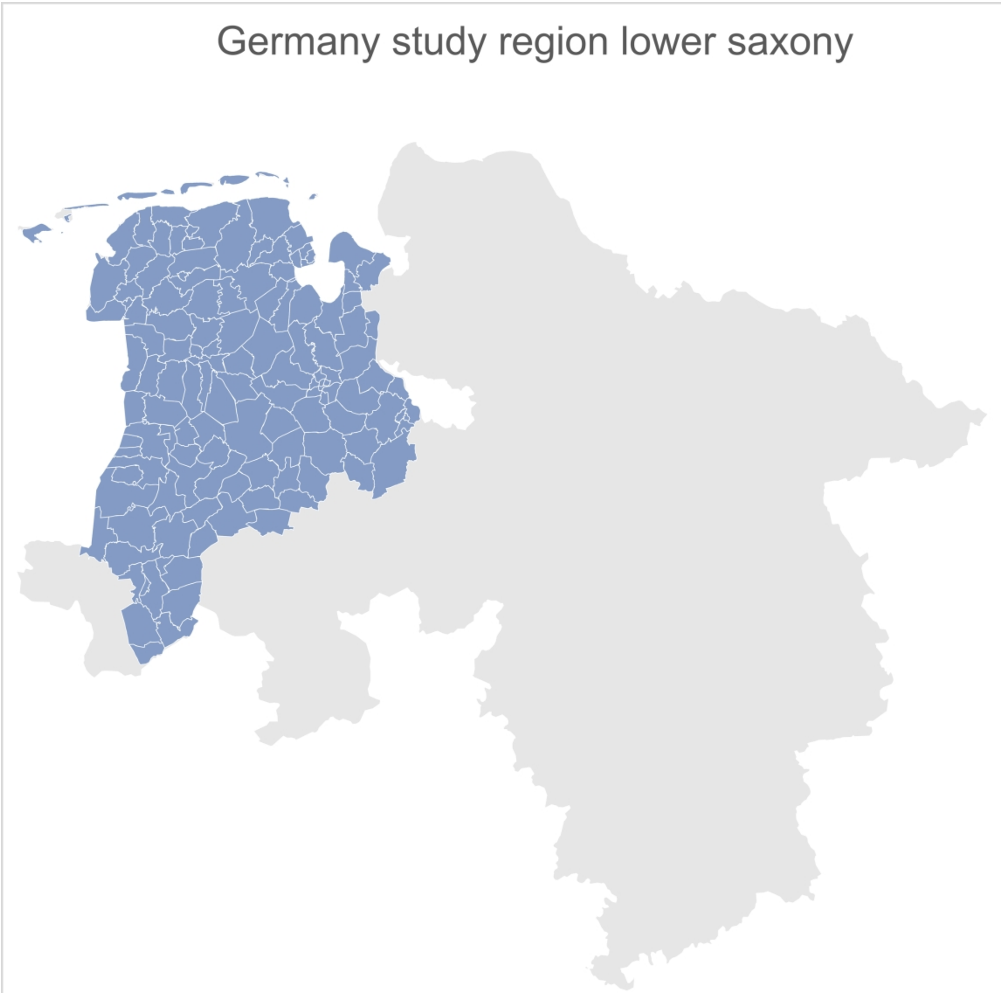

Study region

341x338mm (236 x 236 DPI)

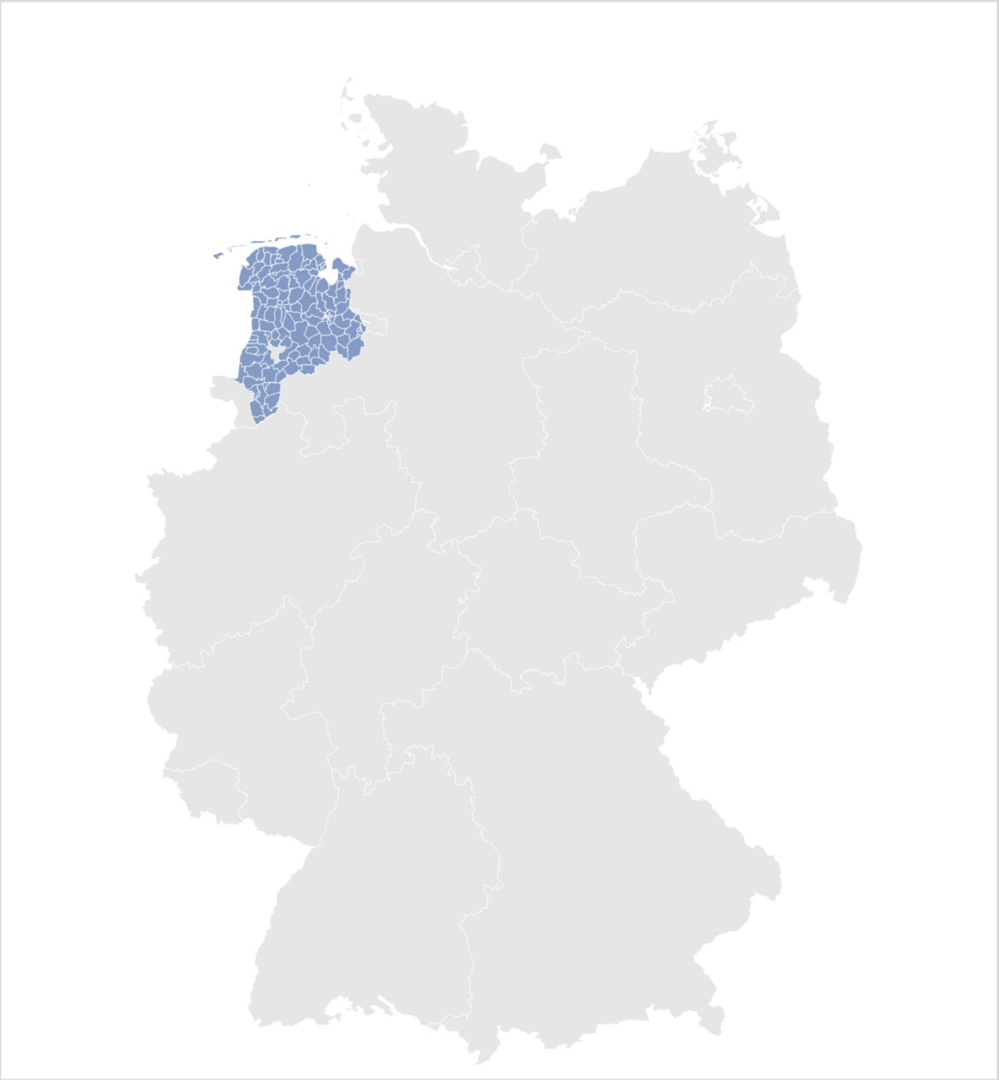

Study region

133x144mm (300 x 300 DPI)

t tests – Means: Difference between two independent means (two groups)

**Analysis:**	A priori: Compute required sample size		
**Input:**	Tail(s)	=	Two
	Effect size d	=	0.3409275
	α err prob	=	0.05
	Power (1–β err prob)	=	0.80
	Allocation ratio N2/N1	=	1
**Output:**	Noncentrality parameter δ	=	2.8216771
	Critical t	=	1.9687238
	Df	=	272

Sample Size Calculation

70x37mm (300 x 300 DPI)

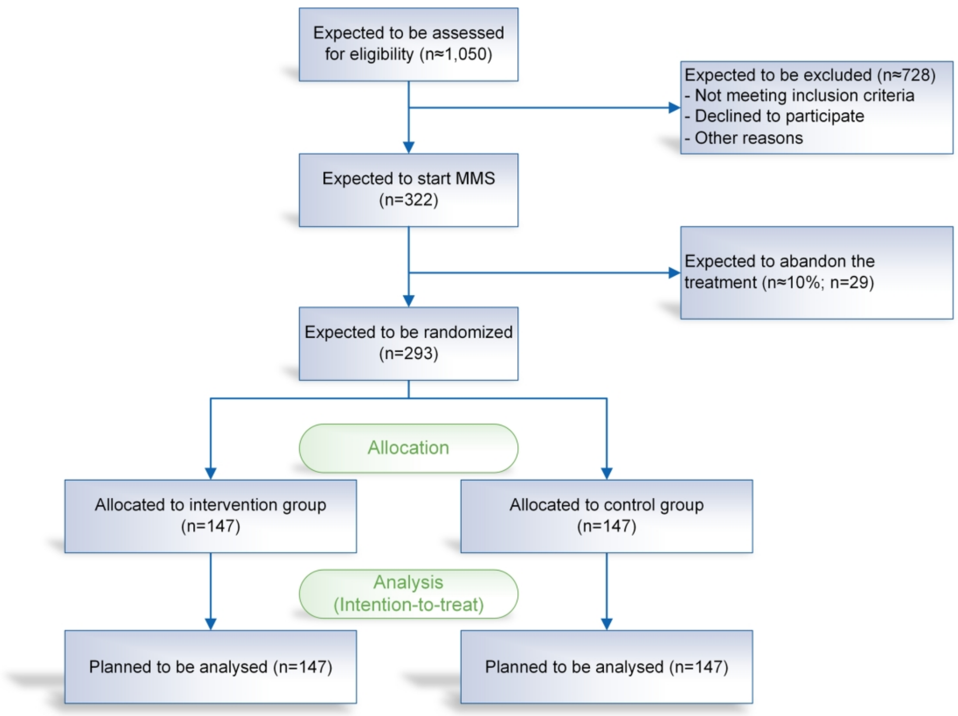

CONSORT Flow Diagram

205x147mm (300 x 300 DPI)

Evaluation of a multimodal pain therapy approach with relapse prophylaxis (MMS-RFP study): study protocol for a cluster randomized controlled trial

Journal:	*BMJ Open*
Manuscript ID	bmjopen-2022-067412.R1
Article Type:	Protocol
Date Submitted by the Author:	28-Mar-2023

Complete List of Authors:	Krueger, Kathrin; Hannover Medical School, Institute for Epidemiology, Social Medicine and Health Systems Research; Center For Health Economics Research<br>Schmetsdorf, Julia; Hannover Medical School, Institute for Epidemiology, Social Medicine and Health Systems Research; Center For Health Economics Research<br>Pavlovic, Maja; AOK Niedersachsen<br>Runde, Werner; Seaside Rehabilitation Hospital Bad Zwischenahn<br>Zechel, Georg; Seaside Rehabilitation Hospital Bad Zwischenahn<br>Hemken, Norbert; Seaside Rehabilitation Hospital Bad Zwischenahn<br>Krauth, Christian; Hannover Medical School, Institute for Epidemiology, Social Medicine and Health Systems Research; Center For Health Economics Research

**Primary Subject Heading**:	Public health
Secondary Subject Heading:	Rehabilitation medicine, Health economics
Keywords:	Back pain < ORTHOPAEDIC & TRAUMA SURGERY, HEALTH ECONOMICS, PAIN MANAGEMENT

SCHOLARONE™ Manuscripts

Evaluation of a multimodal pain therapy approach with relapse prophylaxis (MMS-RFP study): study protocol for a cluster randomized controlled trial

Kathrin Krueger[a,b¶], Julia Schmetsdorf[a,b¶], Maja Pavlovic[c], Werner Runde[d], Georg Zechel[d], Norbert Hemken[d], Christian Krauth[a,b]

a Institute for Epidemiology, Social Medicine, and Health Systems Research, Hannover Medical School, Hannover, Germany

b Center for Health Economics Research (CHERH), Hannover, Germany

c AOK Niedersachsen, Hannover, Germany

d Rehabilitation Center, Outpatient department for orthopedic rehabilitation, Bad Zwischenahn, Germany

Kathrin Krüger and Julia Schmetsdorf contributed equally to this paper.

Keywords: Health economic evaluation, back pain, relapse prevention, interdisciplinary multimodal pain management, early interdisciplinary assessment

Corresponding Author:	Julia Schmetsdorf
Corresponding Author's address:	Carl-Neuberg-Str. 1
	30625 Hannover
	Germany
Corresponding Author's telephone number:	+49 511 532 4449
Corresponding Author's e-mail:	schmetsdorf.julia@mh-hannover.de

Word count: 4,371

For peer review only - http://bmjopen.bmj.com/site/about/guidelines.xhtml

ABSTRACT

Introduction: The need for an interdisciplinary multimodal approach to the treatment of back pain has already been demonstrated by various studies. However, when considering the periods of incapacity to work in the longitudinal course after the multimodal pain therapy (MPT), limits in terms of its sustainable effect become clear. Patients who receive MPT subsequently return to standard outpatient care, which is associated with a risk of relapse. A twelve-month relapse prophylaxis program (RP), intended to follow a four-week MPT, was developed to help patients make the transition to health-conscious and physically active behaviour in everyday life and to identify and prevent impending relapses at an early stage. The evaluation, based on a cluster randomized controlled trial, seeks to provide information on the benefits of early and intensive RP as part of MPT, examine whether it is cost-effective, reduces the days of incapacity to work and increases functional capacity, as well as to examine other parameters.

Methods and analysis: The study population comprises members of a regional statutory health insurance fund in Germany, who are ≤ 62 years old, gainfully employed and have been incapacitated for work for at least 21 days due to a diagnosis of back pain. Over a recruitment period of 24 months, a maximum of 368 individuals can potentially be included in the MPT. The intervention group (IG) and control group (CG) will both receive MPT, after randomization IG will receive RP and CG will receive no further therapy or support as part of the trial. The evaluation is carried out on the following levels: structural, process and results quality. Cost-effectiveness is also assessed by means of a health economic evaluation. In addition to the collection of qualitative and quantitative primary data, claims data from the regional health insurance fund are also included in the analysis.

Ethics and dissemination: This study has received approval by the ethics committee of the Hannover Medical School (reference number 8548_BO_S_2019) and is registered at German Clinical Trials Register DRKS00017654. The study results will be disseminated in national and international journals and conference presentations.

For peer review only - http://bmjopen.bmj.com/site/about/guidelines.xhtml

Word count: 342

For peer review only

For peer review only - http://bmjopen.bmj.com/site/about/guidelines.xhtml

ARTICLE SUMMARY

Strengths and limitations of this study

- A comprehensive evaluation will be provided regarding the ability of this new treatment approach in form of relapse prevention to treat back pain adequately and furthermore support patients in their behavior and motivation to take care of their back health themselves before chronicity occurs. At the same time, it will be examined whether the question if people will be strengthened in their self-efficacy and self-concordance in physical activity to pursue and achieve their health goals in the long term.
- The results will contribute a better understanding of whether the acceptance of the program could be increased by involving and closely accompanying the patients and providing them with concrete training instructions. This highlights high demands on the treatment concept, which are not covered by standard outpatient care.
- The primary outcome is not collected via questionnaires but rather via claims data, so that an information bias can be excluded in this respect.
- As a cluster-randomized study with monthly randomization, a certain degree of contamination, due to personnel changes, for example, is unavoidable, which is a limitation of the methodology. Moreover, the cluster design of this trial entails some potential for selection bias.
- As a limitation for the primary outcome, it should be taken into account that the days of incapacity to work are based on incapacity certificates issued by the treating physician, which are usually only submitted to the health insurance funds for incapacity to work lasting three days or longer. However, this applies to both the intervention and the control group. Furthermore, for back pain, the duration of incapacity to work is generally expected to be greater than two days, and since only days of incapacity to work for back pain (using ICD codes) are used for the primary outcome, the impact is likely to be small.

For peer review only

INTRODUCTION

Back pain has a high prevalence in today's society and is one of the most common complaints in the population. In addition to frequency of occurrence, the main criteria for the assessment of back pain are severity, extent of functional impairment, and prognosis with regards to return to functional and thus work capacity. Data on the prevalence of back pain in Germany include results from several regional and national surveys. The range of the prevalence lies between 30 and 70%, depending on the period considered (point prevalence, 7-day, 3-month, 1-year prevalence) [1]. The lifetime prevalence for back pain in Germany ranges from 74 to 85% [2]. In terms of chronic back pain, the lifetime prevalence lies between 24 and 30% [3]. With regards to incapacity to work, there are various references in the literature [2, 4–6]. 68-86% of patients with acute low back pain return to work within one month [4]. The recurrence rate of low back and neck pain is 47-63%, the relapse rate into inability to work is 33% [1, 5, 7]. Relapse refers to the recurrence of back pain after temporary improvement. Musculoskeletal disorders are among the most expensive diseases in industrialized countries [8]. According to the 2008 Federal Health Report, the costs of non-specific low back pain amounted to 3.6 billion euros [2, 8]. The majority of these costs are due to chronic low back pain and indirect costs resulting from the loss of patients' ability to work. Wenig et al. estimated the average total costs of back pain per patient to be 1,322 € per year [9, 10]. As a cause of premature retirement due to reduced earning capacity, musculoskeletal disorders have taken second place in recent years after mental illness and behavioral disorders [2, 8]. A large percentage of the elderly population suffers from one or more musculoskeletal disorders. However, chronic back pain, reported by about a quarter of women and 17% of men in Germany, often affects younger people in working life [11].

Back pain is usually treated in a monodisciplinary manner, although psychosocial risk factors in particular often hinder recovery and require a multimodal approach with close content-related therapy consultation [12, 13]. Interdisciplinary multimodal treatment is not yet implemented in standard outpatient care.

For peer review only - http://bmjopen.bmj.com/site/about/guidelines.xhtml

Interdisciplinary multimodal pain therapy describes the simultaneous, comprehensive treatment in which various somatic, physical and psychological interventions are integrated according to a predefined treatment plan with an identified therapeutic goal. The treatment is provided in small groups with a maximum of eight patients by a team of therapists consisting of physicians, psychologists or psychotherapists and other disciplines such as physiotherapists and others [14]. MPT is currently considered the gold standard in pain management and treatment for patients with chronic or potentially chronic back pain [15]. The efficacy of interdisciplinary multimodal pain therapy (MPT) in the treatment of chronic or recurrent back pain with risk of chronification has already been proven in various studies [8, 12, 14, 16–20]. The term recurrent describes pain episodes that reoccur after a symptom-free period of at least six months [8]. Chronification moreover, describes the transition from temporary to permanent, or chronic, back pain. After successful MPT, patients usually return to the monodisciplinary treatment of their general practitioner or orthopedist. As a result, the risk of recurrence remains with the end of MPT. The goal of this new approach to the treatment of back pain is to achieve sustainability of MPT by combining MPT with relapse prophylaxis (RP). Thus, what is learned during multimodal treatment in terms of physical activity and lifestyle can be applied to everyday life in the long term.

The intervention examined in this study, the twelve-month relapse prophylaxis (RP) program, immediately follows the four-week MPT. Like the MPT, the RP will be performed by the Rehabilitation Center Bad Zwischenahn on an outpatient basis. The rehabilitation center is completely independent of the evaluating institution. The RP aims to provide prophylaxis against chronifying back pain relapses, but also to support life-integrated physical activity. RP is the all-important link between the curative MPT phase beforehand and the patient's physical activity afterwards. The patient will be assigned to one of three RP paths at the end of the MPT as part of an assessment during the interdisciplinary rounds and team meetings. The paths correspond to RP programs with three different intensities (low, medium and high) for which the patient will be selected based on the severity of his or her complaints (see Figure 1).

For peer review only - http://bmjopen.bmj.com/site/about/guidelines.xhtml

The low intensity level will include the opportunity for telephone consultation and the maintaining of an exercise diary as well as an obligatory telephone conversation after six weeks. After six months and again after one year, a three-hour training session will take place at the Rehabilitation Center with performance tests, examination of the exercise diary and therapeutically supervised training by physiotherapists and orthopedists. In addition, a psychological group session will be conducted by psychologists.

The medium intensity level will additionally include a training session after three months and another obligatory phone call in the fourth and eighth week after the MPT.

In the case of high-intensity RP, a weekly training session (twelve weeks of 90 minutes each) under therapeutic supervision will be scheduled directly after the MPT. Sixteen and twenty weeks post MPT, obligatory telephone calls with the patient will be conducted. The Follow-up training course will be supplemented by another session after nine months. For patients with a particularly high need for follow-up treatment, a one-week training course will be offered after twelve months.

The RP offers the advantage of facilitating the patient's transition into everyday life, accompanying and encouraging progress, as well as enabling early recognition and prevention of relapses. The further development of care thus consists of uninterrupted, seamless and long-term care of back pain patients following multimodal pain therapy. Another goal is to motivate patients during both the MPT phase and the subsequent twelve-month RP phase under multi-professional guidance to adopt a lifelong active lifestyle. While developing good intentions seems easy, turning them into actions is a challenge. For many, the hurdles to a permanently active lifestyle in everyday life are high [21]. RP therefore serves primarily to provide professional support for the patient in the conscious, voluntary translation of goals and motives into results. For patients suffering from back pain, this can be regarded as a secondary prophylaxis against renewed pain, functional impairment and incapacity to work. In this respect, patients at risk of recurrent back pain are on the same level as patients with other

For peer review only - http://bmjopen.bmj.com/site/about/guidelines.xhtml

chronic widespread diseases, such as diabetes or cardiovascular diseases, for which there has long been a consensus on appropriate prevention.

Multimodal Pain Therapy

Employees of the AOK Lower Saxony will contact the potentially eligible insured and refer them to the Rehabilitation Center. Before starting MPT, a comprehensive initial examination will be performed by orthopedists, physiotherapists and psychologists. The MPT program intensity depends on the severity of the back pain. Patients in the standard MPT group will be treated for three to four hours per day, four days a week, while patients in the complex MPT will be treated for five to seven hours per day, five days a week. Two groups of eight patients each will be conducted in parallel. The complex therapy group will provide additional treatment and place more emphasis on psychological and medical intervention aspects, while patients in the standard group will be advised to exercise on their own during these times. Overall, it is expected that patients will benefit from the structured approach of the program.

Modules of the MPT program will be medical lectures and seminars such as social medicine (e.g. payment of wages in the event of illness, professional reintegration), information about the MPT concept and special features of MPT and RP, basic information in different lectures (e.g. structure and function of the spine, muscles, health-disease conception: bio-psychosocial model) and seminars addressing common questions on topics such as obesity and back pain or correlations of symptoms with stress, sports, nutrition, medications, surgery, lifting, and carrying. Another module will consist of medical consultations once a week, alternating between sessions with and without a physiotherapist or psychologist. In addition regular medical consultations will take place for support in case of abnormalities in the course of the disease and for individual treatment (manual therapy, medication etc.) if needed. Moreover, psychological-pain therapy lectures and seminars will be carried out by psychologists. These will educate patients about pain and stress, mindfulness, problematic behavior patterns and setting realistic goals. Psychological training sessions will provide practical implementations in

For peer review only - http://bmjopen.bmj.com/site/about/guidelines.xhtml

form of relaxation techniques, stress management, progressive muscle relaxation and distraction from pain. In addition to individual psychological consultation, regular psychological counseling sessions will be offered. Finally, physiotherapy, occupational therapy, medical training therapy, sports therapy and a module on motivational and volitional aspects will complete the offer.

Figure 1: Treatment Algorithm.

AIMS

The objective of the study is to evaluate the benefit of early and intensive relapse prevention in the context of multimodal pain therapy. The goal is to prevent the chronification of back pain and thus reduce the high direct and indirect costs. To answer this question, outcome, process and cost-effectiveness evaluations will be performed. The aim of outcome evaluation is to examine the intervention effect on patient-relevant outcomes. Process evaluation will examine the extent to which the intervention has been carried out and how the process is perceived and evaluated from the perspective of service providers and patients. Indirect and direct costs will be considered in the health economic evaluation.

From this, the following hypotheses can be derived:

- H_1: The intervention reduces the days of incapacity to work (outcome evaluation)
- H_2: The intervention increases the functional capacity (outcome evaluation)
- H_3: The intervention is perceived positively by the participating patients (process evaluation)
- H_4: The intervention reduces direct and indirect costs (health economic evaluation)

METHODS AND ANALYSIS

Study design

For peer review only - http://bmjopen.bmj.com/site/about/guidelines.xhtml

In order to answer the research questions, outcome, process, and health economic analyses will be carried out. Different research methods will be used for this purpose. The comparative population will be formed by randomizing the groups of participants. The division into the respective groups (intervention group, IG or control group, CG) will be carried out monthly to ensure that the patients of both groups are not able to exchange information with each other. Patients who start an MPT simultaneously in the Rehabilitation Center each form a cluster. These clusters will be randomized to the IG or CG (see Figure 2). The evaluator will inform the Rehabilitation Center monthly via email in the third week of the MPT whether the group is an IG or CG. Up to this point, neither the patient nor the treatment staff will know which group the patient belongs to. The recruitment period will be 24 months.

Figure 2: Study design.

Outcome evaluation

The results will be derived from a cluster-randomized controlled trial (C-RCT). The evaluation design incorporates an IG as well as a control group (CG) and surveys at three different points in time (see Figure 2). Both groups will participate in the MPT program. While IG members will additionally receive relapse prevention modules, the CG will undergo usual care. The primary outcome represents the number of days of incapacity to work within one year after the end of MPT. It is assumed that in the present sample, the average number of 37 days off work per year will decrease in the intervention group (IG) by 40% (or 15 days) compared to the control group (CG). One secondary outcome addresses the functional capacity. It is assumed that the functional capacity in the intervention group will be significantly ($p < 0.05$) higher than in the control group. In addition, patient and provider satisfaction with the intervention will be explored, including barriers, hurdles, and potentials. Direct and indirect costs are another outcome and will be compared between IG and CG.

For peer review only - http://bmjopen.bmj.com/site/about/guidelines.xhtml

Process evaluation

To evaluate the process quality, we will examine the assignment of participants to the standard and the complex program along with the distribution of the IG to low, medium and high intensity RP. In addition, we will consider usage of the modules (e.g., Medical Training Therapy, Psychological Support, Phone Consultation) as well as the app utilization. To examine the quality of the intervention, patients will receive additional questions about their satisfaction with individual MPT and RP modules within the survey. In order to evaluate experiences and satisfaction with the intervention, guided interviews will be conducted. These will be carried out with all physicians, psychologists and physiotherapists involved.

Health economic analysis

The health economic analysis will examine the costs, cost savings and cost-effectiveness of the intervention. Therefore, intervention costs, expenditures of health care utilization, indirect costs due to incapacity to work, and the patient's health-related quality of life will be assessed. For the calculation of indirect costs, the days of incapacity to work are multiplied by the average hourly wage in Germany, taking into account whether the individuals work full-time or part-time. The evaluation will be carried out based on claims data. Incremental cost-effectiveness will be analyzed by considering direct and indirect costs as well as the health-related quality of life of both groups, IG and CG.

Measurements

Various instruments will be used in the course of primary data collection, including standardized questionnaires (Table 1). Prior to the start of MPT, the intervention and control groups will receive a questionnaire to assess the status quo at the beginning of the intervention (T0). The questionnaire will be handed out directly at the Rehabilitation Center or sent by mail in individual cases for T0 and after the four-week MPT program (T1). It will comprise various items on socio-demographic and socio-economic parameters together with health-related behavior (exercise, diet, smoking etc.). Health-related Questionnaires will be the Heaviness of

For peer review only - http://bmjopen.bmj.com/site/about/guidelines.xhtml

Smoking Index (HSI) [22], the AUDIT alcohol consumption questionnaire, an effective brief screening test for problem drinking (AUDIT-C) [23] and the Pain Disability Index (PDI) [24]. In addition, the questionnaire will include items about how often and to what extent the study participants exercise [25], as well as questions about diet and nutrition [26]. In addition, standardized survey instruments will be included to assess functional capacity (Questionnaire for the diagnosis of functional disability caused by backache; FFbH-R) [27] and health-related quality of life (EQ-5D) [28]. A visual analog scale will be used to assess the patient's health-related quality of life from 0-100. IG and CG will receive further questionnaires twelve months post-intervention (T2). All questionnaires except for T0 will include questions regarding patient satisfaction and experience with the intervention. Two weeks after handing out or sending the questionnaire, patients who have not yet responded will receive a reminder to increase the response rate. A second reminder will be sent after an additional ten days. Reminders and the final questionnaires (T2) for the IG will be sent by the Rehabilitation Center, while those for the CG will be sent by the health insurance fund.

Secondary data will be included in the analyses based on claims data of the regional health insurance fund AOK Lower Saxony. These will include inpatient treatment, rehabilitation services, pharmaceuticals and remedies along with days of incapacity to work. Moreover, sociodemographic factors of influence (e.g. age, gender, level of education) together with data regarding health-related quality of life will be included in the analysis.

Table 1. Quantitative and qualitative data sources

Target group	Data source	Data type	Instrument	Parameter
**Patients**	Primary data	Quantitative	Questionnaire	Age, gender, sociodemography, satisfaction, functional capacity, health-related quality of life, impairment

For peer review only - http://bmjopen.bmj.com/site/about/guidelines.xhtml

				due to pain, health behavior, sports activity, nutrition, smoking, alcohol
**Medical Staff, Psychologists**	Primary data	Qualitative	Interview	Motivation, expectations, experiences, hurdles and barriers, suggestions for improvement and satisfaction with the intervention
**Patients**	Secondary data	Quantitative	Claims data	Days of incapacity to work, region, pharmaceuticals, therapeutic products, outpatient and inpatient care

Study population

The study population will comprise members of the regional health insurance fund AOK Lower Saxony, residing in the northwest of Lower Saxony (see Figure 3). The specific inclusion and exclusion criteria are shown in Table 2.

Table 2. Inclusion and exclusion criteria

**Inclusion criteria**	
Membership	AOK Lower Saxony
Region	Northwest of Lower Saxony (see Figure 3)
Sick pay	Entitled
Incapacity to work	At least 21 days during the last six months

For peer review only - http://bmjopen.bmj.com/site/about/guidelines.xhtml

Diagnoses	Back pain (ICD: M40-M54, excluding M45, M46 and M49)
**Exclusion criteria**	
Age	≥ 62 years
Diagnoses	• Mental and behavioral disorders (ICD: F00-F99; six months before)<br>• Malignant neoplasms (ICD: C00-C97; twelve months before incapacity to work)<br>• Injuries (ICD: S00-S99; six months before incapacity to work)<br>• Chronic back pain (duration of the incapacity to work within the last six months is cumulatively longer than twelve weeks)

Figure 3: Study region in Lower Saxony Germany with the study center marked in red.

Study population and participant recruitment

Each month, with the exception of December, two MPT courses with eight participants each can be held at the Rehabilitation Center Bad Zwischenahn. In December, a single course will be held. Over a recruitment period of 24 months, a maximum of 368 individuals could begin an MPT. Potentially eligible individuals will be identified and contacted through health insurance employees. If they are interested, they will be informed and invited to participate in the program. A diagnostic day will then be arranged at the Rehabilitation Center (see Figure 3), where the medical criteria will be reviewed once again.

The risk factors of a possibly low willingness to participate or a high dropout rate of patients were counteracted in advance by the close involvement of the specialized employees of the AOK. For example, the inclusion and exclusion criteria were developed based on their experience. Nevertheless, the development of the case number and the dropout rate will be subject to constant monitoring so that actions can be taken and incentives implemented if necessary.

Sample Size calculation

For peer review only - http://bmjopen.bmj.com/site/about/guidelines.xhtml

For peer review only

The case number calculation was carried out for the primary outcome days of incapacity to work. Currently, the described patient group of AOK-insured persons has an average number of 37 days off work (SD: 58) per year. It is assumed that the average number of 37 days off work per year will decrease in the intervention group (IG) by 40% (or 15 days) compared to the control group. (CG). This results in an effect size of d = 0.3409275. To test with a power of 80% (1 - ß = 0.8) and a significance level of 5% (α error = 0.05), a case number of 137 subjects per group is required for significant proof of the expected effect. The number of cases was calculated using the software G*Power (see Figure 4).

Taking an intraclass correlation coefficient (ICC) of 0.01 and a design effect of 1.1 at an average cluster size of 15.3 (22*16+2*8) into account, the required number of cases is 293 subjects, i.e. 147 subjects per group [29–32]. Considering a drop-out of 10% for patients who drop out during the MPT, i.e., before randomization, results in a required total case number of 322. The available population covers the required case number (see Figure 5).

Figure 4: Sample size calculation.

Figure 5: CONSORT Flow Diagram.

Statistical analysis

Qualitative analysis

The qualitative guided interviews will be documented by means of audio recordings. The recordings will be transcribed without personal information (e.g., name, place of residence). The evaluation of the guided interviews will be based on the qualitative content analysis according to Mayring and carried out with the help of the MAXQDA software [33].

Quantitative analysis

For peer review only - http://bmjopen.bmj.com/site/about/guidelines.xhtml

All patient-related data will be pseudonymized by assigning a unique identification number. A plausibility check of the data will be carried out. Initially, descriptive analysis of the data will be performed. Sample characteristics will be analyzed with regard to their distribution in the two groups by means of the Chi-square test for binary variables and Mann-Whitney-U-test for continuous variables.

Primary Outcome

The data will be analyzed by reference to the primary outcome (days of incapacity to work), which is carried out by intention-to-treat analysis. Patients are analyzed according to their original group assignment.

To test the primary hypothesis H_1 "The intervention reduces the number of days of incapacity to work", an analysis of covariances (ANCOVA) with cluster adjustment will be performed, using a significance level of 0.05. Covariables included into the model will be age, gender and comorbidities (e.g. depression). In addition, a hypothesis test (two-sided t-test with a significance level of 0.05) will be performed.

Secondary outcomes

Data for secondary outcome variables will be obtained via questionnaires and claims data. The Markov Chain Monte Carlo approach (MCMC) in SPSS will be used as imputation method to replace missing values [34–36]. To test the hypothesis H_2 "The intervention increases functional capacity" and H_4 "The intervention reduces direct and indirect costs", hypothesis tests (two-sided t-test with a significance level of 0.05) will be performed. The third hypothesis H_3 "The intervention is perceived positively by the participating patients" will be tested by conducting a pencil-paper questionnaire with the patients. The analyses are carried out using the software program SPSS.

Patient and Public Involvement

For peer review only - http://bmjopen.bmj.com/site/about/guidelines.xhtml

No patients were involved in the design and conduct of the study, choice of outcome measures and recruitment to the study. The results of the study will be provided to patients at their request.

ETHICS AND DISSEMINATION

Ethical and scientific standards in the currently valid version will be taken into account when conducting the study, especially the Memorandum on Good Scientific Practice of the German Research Foundation [37]. In particular, analysis of secondary data will be performed according to the Good Practice of Secondary Data Analysis guidelines, whereas the primary data collection will be conducted in accordance with the STROBE (Strengthening the Reporting of Observational Studies in Epidemiology) statement [38]. In addition, the methods of health economic evaluation in health services research and the Hannover Consensus will be applied [39]. Preliminary to the intervention, a concrete analysis plan is defined. Moreover, the questionnaires developed for the MPT and RP will be pre-tested and all data will be subjected to a validation process. In addition, participation in the intervention is voluntary for the insured, so there is no disadvantage if people do not want to participate. This study has received approval by the ethics committee of the Hannover Medical School (reference number 8548_BO_S_2019) and is registered at German Clinical Trials Register DRKS00017654. Each participating patient will sign a contract for participation in the program, and we will obtain written informed consent. Furthermore, all study participants will be comprehensively informed about the use of the collected data and the purpose and procedure of the project. All transmitted data will be pseudonymized and is accessible only to project members. The delivered data will be stored on a secure and encrypted network drive. Access to the files is restricted to the project staff. No data will be passed on to third parties. Furthermore, the information obtained will be used exclusively for research purposes and no information will be passed on that enables conclusions about individual persons. All data collected and processed will be stored on a secure and encrypted hard disk after the end of the study and deleted ten years after (by 31 March 2033 at the latest).

For peer review only - http://bmjopen.bmj.com/site/about/guidelines.xhtml

The study results will be disseminated in national and international journals and conference presentations. The Innovation Fund (G-BA) will publish the study outcomes in a results report. Additionally, the health insurance fund AOK Lower Saxony will make the results available via its homepage and member journals.

For peer review only

For peer review only - http://bmjopen.bmj.com/site/about/guidelines.xhtml

Author contributions

KK and CK conceived, designed and obtained funding for the research. The first draft of this manuscript was produced by KK and JS. All authors have provided input to, reviewed, edited and approved the final version. Conceptualization: KK, CK, WR, GZ. Funding acquisition: CK, MP, NH. Methodology: KK, CK. Project administration: MP. Supervision: CK. Visualization: JS, KK. Writing - original draft: JS, KK. Writing - review & editing: JS, KK, CK, MP, WR, GZ, NH.

Funding statement

This project is funded by the Federal Joint Committee (G-BA), Innovation Fund, (funding code 01NVF18011). The funding company had no role in study design, data collection and analysis, decision to publish, or preparation of the manuscript.

Competing interests

The authors declare that they have no competing interest.

Abbreviations

AOK	Allgemeine Ortskrankenkasse (Regional Statutory Health Insurance Fund)
CG	Control group
IG	Intervention group
RP	Relapse prevention
LBP	Low Back Pain
MPT	Multimodal pain therapy

Appendix

Not available.

Acknowledgements

Not applicable.

For peer review only

REFERENCES

1 Casser H-R, Seddigh S, Rauschmann M. Acute Lumbar Back Pain. *Dtsch Arztebl Int* 2016;113(13):223–34.

2 Raspe H-H, ed. Rückenschmerzen. Berlin: Robert-Koch-Institut (RKI) 2012.

3 Neuhauser H, Ellert U, Ziese T. Chronische Rückenschmerzen in der Allgemeinbevölkerung in Deutschland 2002/2003: Prävalenz und besonders betroffene Bevölkerungsgruppen. *Gesundheitswesen* 2005;67(10):685–93.

4 Pengel LHM, Herbert RD, Maher CG, et al. Acute low back pain: systematic review of its prognosis. *BMJ* 2003;327(7410):323.

5 Mehling WE, Gopisetty V, Bartmess E, et al. The prognosis of acute low back pain in primary care in the United States: a 2-year prospective cohort study. *Spine (Phila Pa 1976)* 2012;37(8):678–84.

6 Hestbaek L, Leboeuf-Yde C, Manniche C. Low back pain: what is the long-term course? A review of studies of general patient populations. *Eur Spine J* 2003;12(2):149–65. doi:10.1007/s00586-002-0508-5 [published Online First: 28 January 2003].

7 Enthoven P, Skargren E, Oberg B. Clinical course in patients seeking primary care for back or neck pain: a prospective 5-year follow-up of outcome and health care consumption with subgroup analysis. *Spine (Phila Pa 1976)* 2004;29(21):2458–65.

8 Bundesärztekammer (BÄK), Kassenärztliche Bundesvereinigung (KBV), Arbeitsgemeinschaft der Wissenschaftlichen Medizinischen Fachgesellschaften (AWMF). Nationale VersorgungsLeitlinie Nicht-spezifischer Kreuzschmerz – Langfassung, 2. Auflage. Version 1 2017.

9 Wenig CM, Schmidt CO, Kohlmann T, et al. Costs of back pain in Germany. *Eur J Pain* 2009;13(3):280–86. doi:10.1016/j.ejpain.2008.04.005 [published Online First: 3 June 2008].

10 Schmidt CO, Raspe H, Pfingsten M, et al. Back pain in the German adult population: prevalence, severity, and sociodemographic correlates in a multiregional survey. *Spine (Phila Pa 1976)* 2007;32(18):2005–11.

11 Robert Koch-Institut. Gesundheit in Deutschland: RKI-Bib1 (Robert Koch-Institut) 2016.

12 Pfingsten M, Arnold B, Böger A, et al. Sektorenübergreifende interdisziplinäre multimodale Schmerztherapie Empfehlungen zu Struktur- und Prozessparametern der

For peer review only - http://bmjopen.bmj.com/site/about/guidelines.xhtml

Ad-hoc-Kommission „Interdisziplinäre Multimodale Schmerztherapie“ der Deutschen Schmerzgesellschaft e. V. *Schmerz* 2019;33(3):191–203.

13 Pfingsten M. Chronischer Rückenschmerz--Interdisziplinäre Diagnostik und Therapie. *Anästhesiol Intensivmed Notfallmed Schmerzther* 2009;44(1):40-5; quiz 46. doi:10.1055/s-0028-1128185 [published Online First: 29 December 2008].

14 Arnold B, Brinkschmidt T, Casser H-R, et al. Multimodale Schmerztherapie: Konzepte und Indikation. *Schmerz* 2009;23(2):112–20.

15 Behrendt S, Kulas H, Marschall U, et al. Which Back Pain Patients Benefit From Multidisciplinary Pain Management? A Comparative Cost Analysis Using Claims Data of Statutory Health Insurance. *Gesundheitswesen* 2016;78(S 01):e120-e127. doi:10.1055/s-0042-110401 [published Online First: 21 July 2016].

16 Michaelson P, Sjölander P, Johansson H. Factors predicting pain reduction in chronic back and neck pain after multimodal treatment. *Clin J Pain* 2004;20(6):447–54.

17 Bergström G, Jensen IB, Bodin L, et al. The impact of psychologically different patient groups on outcome after a vocational rehabilitation program for long-term spinal pain patients. *Pain* 2001;93(3):229–37.

18 Kamper SJ, Apeldoorn AT, Chiarotto A, et al. Multidisciplinary biopsychosocial rehabilitation for chronic low back pain: Cochrane systematic review and meta-analysis. *BMJ* 2015;350:h444. doi:10.1136/bmj.h444 [published Online First: 18 February 2015].

19 Jensen IB, Busch H, Bodin L, et al. Cost effectiveness of two rehabilitation programmes for neck and back pain patients: A seven year follow-up. *Pain* 2009;142(3):202–08. doi:10.1016/j.pain.2008.12.015 [published Online First: 13 February 2009].

20 Nicholas MK, Asghari A, Sharpe L, et al. Cognitive exposure versus avoidance in patients with chronic pain: adherence matters. *Eur J Pain* 2014;18(3):424–37. doi:10.1002/j.1532-2149.2013.00383.x [published Online First: 13 August 2013].

21 Fuchs R, Goehner W, Seelig H. Long-term effects of a psychological group intervention on physical exercise and health: the MoVo concept. *J Phys Act Health* 2011;8(6):794–803.

22 Deutsche Version des Fagerström-Test for Nicotine Dependence (FTND-G) und des Heaviness of Smoking Index (HSI-G) 2003.

23 Bush K, Kivlahan DR, McDonell MB, et al. The AUDIT alcohol consumption questions (AUDIT-C): an effective brief screening test for problem drinking. Ambulatory Care

For peer review only

Quality Improvement Project (ACQUIP). Alcohol Use Disorders Identification Test. *Archives of internal medicine* 1998;158(16):1789–95. https://pubmed.ncbi.nlm.nih.gov/9738608/.

24 Dillmann U, Nilges P, Saile H, et al. Behinderungseinschätzung bei chronischen Schmerzpatienten. *Schmerz* 1994;8(2):100–10.

25 Dlugosch GE, Krieger W. Fragebogen zur Erfassung des Gesundheitsverhaltens: Swets Test Services 1995.

26 Keller S. Zur Validität des Transtheoretischen Modells eine Untersuchung zur Veränderung des Ernährungsverhaltens: Philipps-Universität Marburg 1998.

27 Kohlmann T, Raspe H. Der Funktionsfragebogen Hannover zur alltagsnahen Diagnostik der Funktionsbeeinträchtigung durch Rückenschmerzen (FFbH-R). *Rehabilitation* 1996;35(1):I–VIII.

28 J.-Matthias Graf v. d. Schulenburg, C. Claes, W. Greiner, A. Uber. Die deutsche Version des EuroQol-Fragebogens. *Zeitschrift für Gesundheitswissenschaften* 1998;6(1):3–20.

29 Eldridge SM, Ashby D, Kerry S. Sample size for cluster randomized trials: effect of coefficient of variation of cluster size and analysis method. *Int J Epidemiol* 2006;35(5):1292–300. doi:10.1093/ije/dyl129 [published Online First: 30 August 2006].

30 Dreyhaupt J, Mayer B, Kaluscha R, et al. Cluster-randomisierte Studien: Methodische und praktische Aspekte. *Rehabilitation* 2020;59(1):54–61. doi:10.1055/a-0801-5697 [published Online First: 23 January 2019].

31 Rutterford C, Copas A, Eldridge S. Methods for sample size determination in cluster randomized trials. *Int J Epidemiol* 2015;44(3):1051–67. doi:10.1093/ije/dyv113 [published Online First: 13 July 2015].

32 Lorenz E, Köpke S, Pfaff H, et al. Cluster-Randomized Studies. *Dtsch Arztebl Int* 2018;115(10):163–68.

33 Mayring P. Qualitative Inhaltsanalyse: Grundlagen und Techniken, 11th edn. Weinheim: Beltz 2010.

34 Li K-H. Imputation using markov chains. *Journal of Statistical Computation and Simulation* 1988;30(1):57–79.

35 Rubin BD. Multiple imputation for survey nonresponse 1987.

For peer review only - http://bmjopen.bmj.com/site/about/guidelines.xhtml

For peer review only

36 Mayer B. Fehlende Werte in klinischen Verlaufsstudien - der Umgang mit Studienabbrechern 2011.

37 Vorschläge zur Sicherung guter wissenschaftlicher Praxis: Empfehlungen der Kommission "Selbstkontrolle in der Wissenschaft" ; Denkschrift. Weinheim: Wiley-VCH 2013.

38 Graf von der Schulenburg J-M, Greiner W, Jost F, et al. German recommendations on health economic evaluation: third and updated version of the Hanover Consensus. *Value Health* 2008;11(4):539–44. doi:10.1111/j.1524-4733.2007.00301.x [published Online First: 11 January 2008].

39 Workgroup GPGI. Gute Praxis Gesundheitsinformation. *Z Evid Fortbild Qual Gesundhwes* 2016;110-111:85–92. doi:10.1016/j.zefq.2015.11.005 [published Online First: 13 January 2016].

For peer review only

For peer review only - http://bmjopen.bmj.com/site/about/guidelines.xhtml

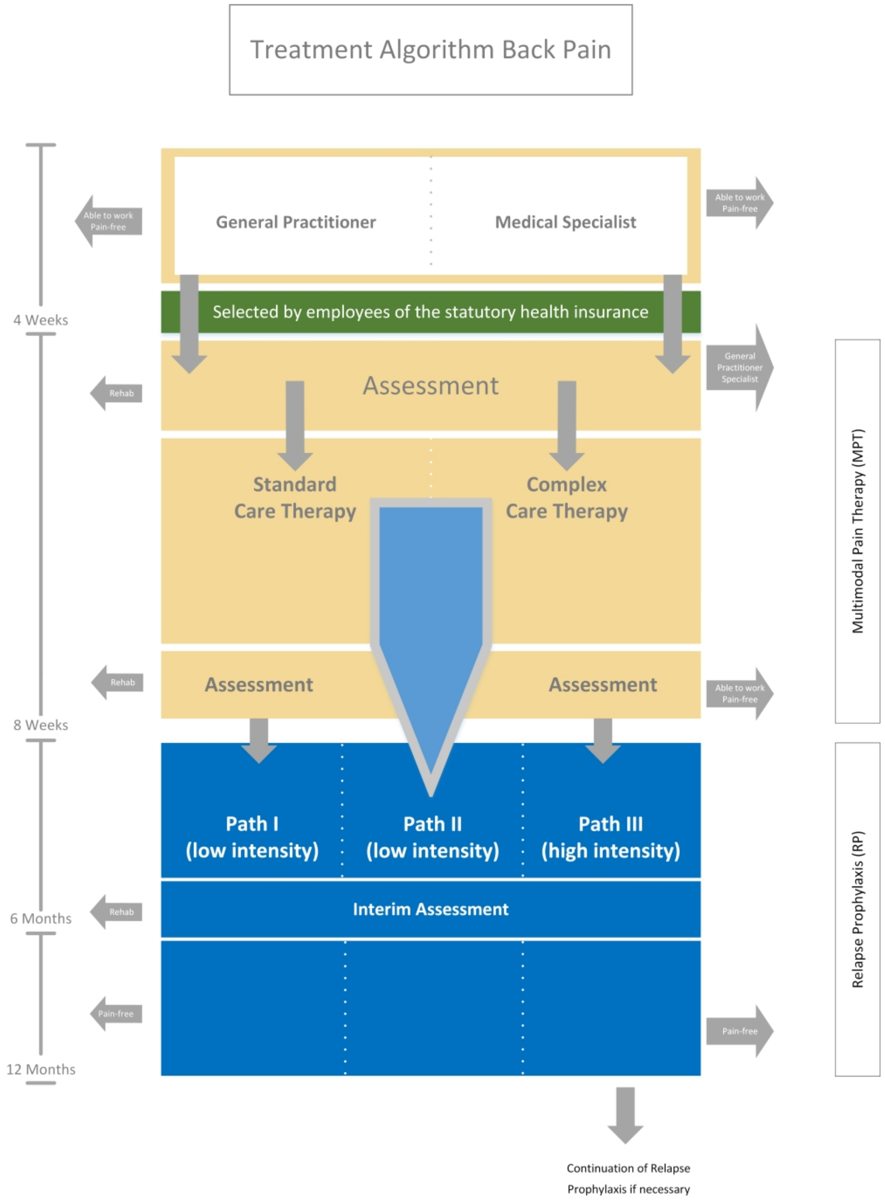

Treatment Algorithm

415x565mm (236 x 236 DPI)

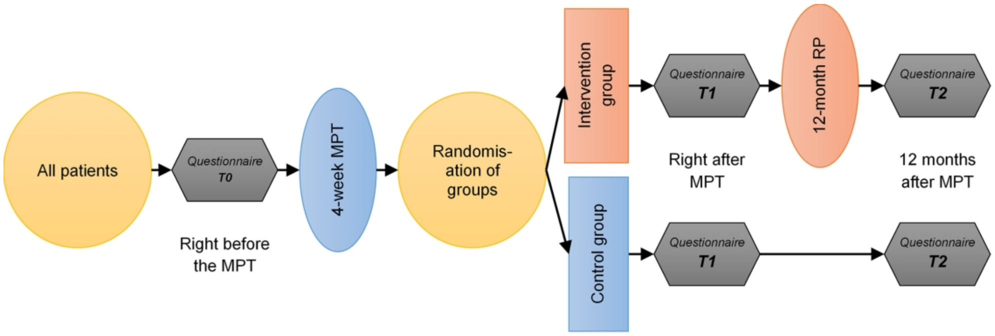

Study design

190x66mm (300 x 300 DPI)

Germany study region lower saxony

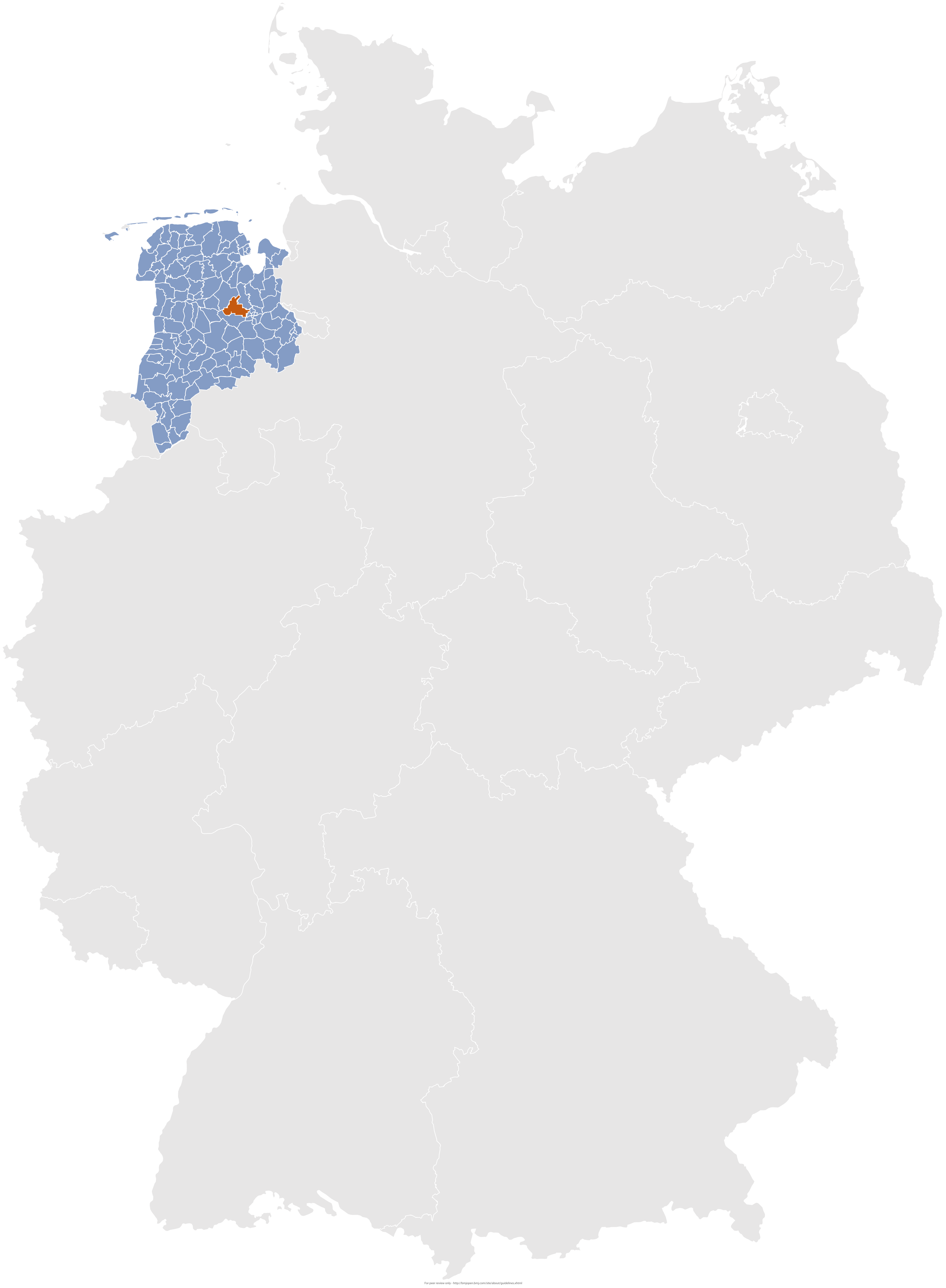

t tests – Means: Difference between two independent means (two groups)

**Analysis:**	A priori: Compute required sample size		
**Input:**	Tail(s)	=	Two
	Effect size d	=	0.3409275
	α err prob	=	0.05
	Power (1–β err prob)	=	0.80
	Allocation ratio N2/N1	=	1
**Output:**	Noncentrality parameter δ	=	2.8216771
	Critical t	=	1.9687238
	Df	=	272

Sample Size Calculation

70x37mm (300 x 300 DPI)

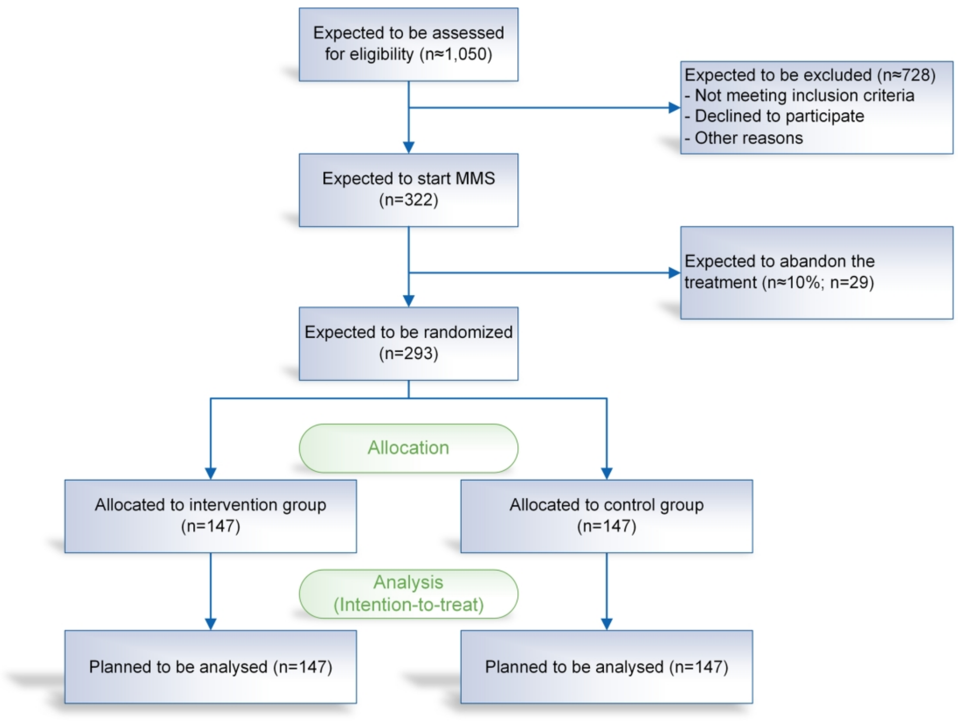

CONSORT Flow Diagram

205x147mm (300 x 300 DPI)

BMJ Open

Evaluation of a multimodal pain therapy approach with relapse prophylaxis for back pain (MMS-RFP study): study protocol for a cluster randomized controlled trial

Journal:	*BMJ Open*
Manuscript ID	bmjopen-2022-067412.R2
Article Type:	Protocol
Date Submitted by the Author:	01-Jun-2023

Complete List of Authors:	Krueger, Kathrin; Hannover Medical School, Institute for Epidemiology, Social Medicine and Health Systems Research; Center For Health Economics Research<br>Schmetsdorf, Julia; Hannover Medical School, Institute for Epidemiology, Social Medicine and Health Systems Research; Center For Health Economics Research<br>Pavlovic, Maja; AOK Niedersachsen<br>Runde, Werner; Seaside Rehabilitation Hospital Bad Zwischenahn<br>Zechel, Georg; Seaside Rehabilitation Hospital Bad Zwischenahn<br>Hemken, Norbert; Seaside Rehabilitation Hospital Bad Zwischenahn<br>Krauth, Christian; Hannover Medical School, Institute for Epidemiology, Social Medicine and Health Systems Research; Center For Health Economics Research

**Primary Subject Heading**:	Public health
Secondary Subject Heading:	Rehabilitation medicine, Health economics
Keywords:	Back pain < ORTHOPAEDIC & TRAUMA SURGERY, HEALTH ECONOMICS, PAIN MANAGEMENT

SCHOLARONE™
Manuscripts

Evaluation of a multimodal pain therapy approach with relapse prophylaxis for back pain (MMS-RFP study): study protocol for a cluster randomized controlled trial

Kathrin Krueger[a,b¶], Julia Schmetsdorf[a,b¶], Maja Pavlovic[c], Werner Runde[d], Georg Zechel[d], Norbert Hemken[d], Christian Krauth[a,b]

a Institute for Epidemiology, Social Medicine, and Health Systems Research, Hannover Medical School, Hannover, Germany

b Center for Health Economics Research (CHERH), Hannover, Germany

c AOK Niedersachsen, Hannover, Germany

d Rehabilitation Center, Outpatient department for orthopedic rehabilitation, Bad Zwischenahn, Germany

Kathrin Krüger and Julia Schmetsdorf contributed equally to this paper.

Keywords: Health economic evaluation, back pain, relapse prevention, interdisciplinary multimodal pain management, early interdisciplinary assessment

Corresponding Author: Julia Schmetsdorf

Corresponding Author's address: Carl-Neuberg-Str. 1
30625 Hannover
Germany

Corresponding Author's telephone number: +49 511 532 4449

Corresponding Author's e-mail: schmetsdorf.julia@mh-hannover.de

Word count: 4,435

For peer review only

For peer review only - http://bmjopen.bmj.com/site/about/guidelines.xhtml

ABSTRACT

Introduction: The need for an interdisciplinary multimodal approach to the treatment of back pain has already been demonstrated by various studies. However, when considering the periods of incapacity to work in the longitudinal course after the multimodal pain therapy (MPT), limits in terms of its sustainable effect become clear. Patients who receive MPT subsequently return to standard outpatient care, which is associated with a risk of relapse. A twelve-month relapse prophylaxis program (RP), intended to follow a four-week MPT, was developed to help patients make the transition to health-conscious and physically active behavior in everyday life and to identify and prevent impending relapses at an early stage. The evaluation, based on a cluster randomized controlled trial, seeks to provide information on the benefits of early and intensive RP as part of MPT, examine whether it is cost-effective, reduces the days of incapacity to work and increases functional capacity, as well as to examine other parameters.

Methods and analysis: The study population comprises members of a regional statutory health insurance fund in Germany, who are ≤ 62 years old, gainfully employed and have been incapacitated for work for at least 21 days due to a diagnosis of back pain. Over a recruitment period of 24 months, a maximum of 368 individuals can potentially be included in the MPT. The intervention group (IG) and control group (CG) will both receive MPT, after randomization IG will receive RP and CG will receive no further therapy or support as part of the trial. The evaluation is carried out on the following levels: structural, process and results quality. Cost-effectiveness is also assessed by means of a health economic evaluation. In addition to the collection of qualitative and quantitative primary data, claims data from the regional health insurance fund are also included in the analysis.

Ethics and dissemination: This study has received approval by the ethics committee of the Hannover Medical School (reference number 8548_BO_S_2019) and is registered at German Clinical Trials Register DRKS00017654. The study results will be disseminated in national and international journals and conference presentations.

For peer review only - http://bmjopen.bmj.com/site/about/guidelines.xhtml

Word count: 342

For peer review only - http://bmjopen.bmj.com/site/about/guidelines.xhtml

ARTICLE SUMMARY

Strengths and limitations of this study

- A comprehensive evaluation will be provided regarding the ability of this new treatment approach in form of relapse prevention to treat back pain adequately and furthermore support patients in their behavior and motivation to take care of their back health themselves before chronicity occurs.
- The results will contribute a better understanding of whether the acceptance of the program could be increased by involving and closely accompanying the patients and providing them with concrete training instructions, which highlights high demands on the treatment concept that are not covered by standard outpatient care.
- The primary outcome is not collected via questionnaires but rather via claims data, so that an information bias can be excluded in this respect.
- As a cluster-randomized study with monthly randomization, a certain degree of contamination, due to personnel changes, for example, is unavoidable, which is a limitation of the methodology.
- As a limitation for the primary outcome, it should be taken into account that the days of incapacity to work are based on incapacity certificates issued by the treating physician, which are usually only submitted to the health insurance funds for incapacity to work lasting three days or longer, but since a period of incapacity to work of more than two days is usually to be expected in the case of back pain, the impact is likely to be small.

For peer review only - http://bmjopen.bmj.com/site/about/guidelines.xhtml

INTRODUCTION

Back pain has a high prevalence in today's society and is one of the most common complaints in the population. In addition to frequency of occurrence, the main criteria for the assessment of back pain are severity, extent of functional impairment, and prognosis with regards to return to functional and thus work capacity. Data on the prevalence of back pain in Germany include results from several regional and national surveys. The range of the prevalence lies between 30 and 70%, depending on the period considered (point prevalence, 7-day, 3-month, 1-year prevalence) [1]. The lifetime prevalence for back pain in Germany ranges from 74 to 85% [2]. In terms of chronic back pain, the lifetime prevalence lies between 24 and 30% [3]. With regards to incapacity to work, there are various references in the literature [2, 4–6]. 68-86% of patients with acute low back pain return to work within one month [4]. The recurrence rate of low back and neck pain is 47-63%, the relapse rate into inability to work is 33% [1, 5, 7]. Relapse refers to the recurrence of back pain after temporary improvement. Musculoskeletal disorders are among the most expensive diseases in industrialized countries [8]. According to the 2008 Federal Health Report, the costs of non-specific low back pain amounted to 3.6 billion euros [2, 8]. The majority of these costs are due to chronic low back pain and indirect costs resulting from the loss of patients' ability to work. Wenig et al. estimated the average total costs of back pain per patient to be 1,322 € per year [9, 10]. As a cause of premature retirement due to reduced earning capacity, musculoskeletal disorders have taken second place in recent years after mental illness and behavioral disorders [2, 8]. A large percentage of the elderly population suffers from one or more musculoskeletal disorders. However, chronic back pain, reported by about a quarter of women and 17% of men in Germany, often affects younger people in working life [11].

Back pain is usually treated in a monodisciplinary manner, although psychosocial risk factors in particular often hinder recovery and require a multimodal approach with close content-related therapy consultation [12, 13]. Interdisciplinary multimodal treatment is not yet implemented in standard outpatient care.

For peer review only - http://bmjopen.bmj.com/site/about/guidelines.xhtml

Interdisciplinary multimodal pain therapy describes the simultaneous, comprehensive treatment in which various somatic, physical and psychological interventions are integrated according to a predefined treatment plan with an identified therapeutic goal. The treatment is provided in small groups with a maximum of eight patients by a team of therapists consisting of physicians, psychologists or psychotherapists and other disciplines such as physiotherapists and others [14]. MPT is currently considered the gold standard in pain management and treatment for patients with chronic or potentially chronic back pain [15]. The efficacy of interdisciplinary multimodal pain therapy (MPT) in the treatment of chronic or recurrent back pain with risk of chronification has already been proven in various studies [8, 13, 14, 16–20]. The term recurrent describes pain episodes that reoccur after a symptom-free period of at least six months [8]. Chronification moreover, describes the transition from temporary to permanent, or chronic, back pain. After successful MPT, patients usually return to the monodisciplinary treatment of their general practitioner or orthopedist. As a result, the risk of recurrence remains with the end of MPT. The goal of this new approach to the treatment of back pain is to achieve sustainability of MPT by combining MPT with relapse prophylaxis (RP). Thus, what is learned during multimodal treatment in terms of physical activity and lifestyle can be applied to everyday life in the long term.

The intervention examined in this study, the twelve-month relapse prophylaxis (RP) program, immediately follows the four-week MPT. Like the MPT, the RP will be performed by the Rehabilitation Center Bad Zwischenahn on an outpatient basis. The rehabilitation center is completely independent of the evaluating institution. The RP aims to provide prophylaxis against chronifying back pain relapses, but also to support life-integrated physical activity. RP is the all-important link between the curative MPT phase beforehand and the patient's physical activity afterwards. The patient will be assigned to one of three RP paths at the end of the MPT as part of an assessment during the interdisciplinary rounds and team meetings. The paths correspond to RP programs with three different intensities (low, medium and high) for which the patient will be selected based on the severity of his or her complaints (see Figure 1).

For peer review only - http://bmjopen.bmj.com/site/about/guidelines.xhtml

The low intensity level will include the opportunity for telephone consultation and the maintaining of an exercise diary as well as an obligatory telephone conversation after six weeks. After six months and again after one year, a three-hour training session will take place at the Rehabilitation Center with performance tests, examination of the exercise diary and therapeutically supervised training by physiotherapists and orthopedists. In addition, a psychological group session will be conducted by psychologists.

The medium intensity level will additionally include a training session after three months and another obligatory phone call in the fourth and eighth week after the MPT.

In the case of high-intensity RP, a weekly training session (twelve weeks of 90 minutes each) under therapeutic supervision will be scheduled directly after the MPT. Sixteen and twenty weeks post MPT, obligatory telephone calls with the patient will be conducted. The Follow-up training course will be supplemented by another session after nine months. For patients with a particularly high need for follow-up treatment, a one-week training course will be offered after twelve months.

The RP offers the advantage of facilitating the patient's transition into everyday life, accompanying and encouraging progress, as well as enabling early recognition and prevention of relapses. The further development of care thus consists of uninterrupted, seamless and long-term care of back pain patients following multimodal pain therapy. Another goal is to motivate patients during both the MPT phase and the subsequent twelve-month RP phase under multi-professional guidance to adopt a lifelong active lifestyle. While developing good intentions seems easy, turning them into actions is a challenge. For many, the hurdles to a permanently active lifestyle in everyday life are high [21]. RP therefore serves primarily to provide professional support for the patient in the conscious, voluntary translation of goals and motives into results. For patients suffering from back pain, this can be regarded as a secondary prophylaxis against renewed pain, functional impairment and incapacity to work. In this respect, patients at risk of recurrent back pain are on the same level as patients with other

For peer review only - http://bmjopen.bmj.com/site/about/guidelines.xhtml

chronic widespread diseases, such as diabetes or cardiovascular diseases, for which there has long been a consensus on appropriate prevention.

Multimodal Pain Therapy

Employees of the AOK Lower Saxony will contact the potentially eligible insured and refer them to the Rehabilitation Center. Before starting MPT, a comprehensive initial examination will be performed by orthopedists, physiotherapists and psychologists. The MPT program intensity depends on the severity of the back pain. Patients in the standard MPT group will be treated for three to four hours per day, four days a week, while patients in the complex MPT will be treated for five to seven hours per day, five days a week. Two groups of eight patients each will be conducted in parallel. The complex therapy group will provide additional treatment and place more emphasis on psychological and medical intervention aspects, while patients in the standard group will be advised to exercise on their own during these times. Overall, it is expected that patients will benefit from the structured approach of the program.

Modules of the MPT program will be medical lectures and seminars such as social medicine (e.g. payment of wages in the event of illness, professional reintegration), information about the MPT concept and special features of MPT and RP, basic information in different lectures (e.g. structure and function of the spine, muscles, health-disease conception: bio-psychosocial model) and seminars addressing common questions on topics such as obesity and back pain or correlations of symptoms with stress, sports, nutrition, medications, surgery, lifting, and carrying. Another module will consist of medical consultations once a week, alternating between sessions with and without a physiotherapist or psychologist. In addition regular medical consultations will take place for support in case of abnormalities in the course of the disease and for individual treatment (manual therapy, medication etc.) if needed. Moreover, psychological-pain therapy lectures and seminars will be carried out by psychologists. These will educate patients about pain and stress, mindfulness, problematic behavior patterns and setting realistic goals. Psychological training sessions will provide practical implementations in

For peer review only - http://bmjopen.bmj.com/site/about/guidelines.xhtml

form of relaxation techniques, stress management, progressive muscle relaxation and distraction from pain. In addition to individual psychological consultation, regular psychological counseling sessions will be offered. Finally, physiotherapy, occupational therapy, medical training therapy, sports therapy and a module on motivational and volitional aspects will complete the offer.

Figure 1: Treatment Algorithm.

AIMS

The objective of the study is to evaluate the benefit of early and intensive relapse prevention in the context of multimodal pain therapy. The goal is to prevent the chronification of back pain and thus reduce the high direct and indirect costs. To answer this question, outcome, process and cost-effectiveness evaluations will be performed. The aim of outcome evaluation is to examine the intervention effect on patient-relevant outcomes. Process evaluation will examine the extent to which the intervention has been carried out and how the process is perceived and evaluated from the perspective of service providers and patients. Indirect and direct costs will be considered in the health economic evaluation.

From this, the following hypotheses can be derived:

- H_1: The intervention reduces the days of incapacity to work (outcome evaluation)
- H_2: The intervention increases the functional capacity (outcome evaluation)
- H_3: The intervention is perceived positively by the participating patients (process evaluation)
- H_4: The intervention reduces direct and indirect costs (health economic evaluation)

METHODS AND ANALYSIS

Study design

For peer review only - http://bmjopen.bmj.com/site/about/guidelines.xhtml

For peer review only

In order to answer the research questions, outcome, process, and health economic analyses will be carried out. Different research methods will be used for this purpose. The comparative population will be formed by randomizing the groups of participants. The division into the respective groups (intervention group, IG or control group, CG) will be carried out monthly to ensure that the patients of both groups are not able to exchange information with each other. Patients who start an MPT simultaneously in the Rehabilitation Center each form a cluster. These clusters will be randomized to the IG or CG (see Figure 2). The evaluator will inform the Rehabilitation Center monthly via email in the third week of the MPT whether the group is an IG or CG. Up to this point, neither the patient nor the treatment staff will know which group the patient belongs to. The recruitment period will be 24 months.

Figure 2: Study design.

Outcome evaluation

The results will be derived from a cluster-randomized controlled trial (C-RCT). The evaluation design incorporates an IG as well as a control group (CG) and surveys at three different points in time (see Figure 2). Both groups will participate in the MPT program. While IG members will additionally receive relapse prevention modules, the CG will undergo usual care. The primary outcome represents the number of days of incapacity to work within one year after the end of MPT. It is assumed that in the present sample, the average number of 37 days off work per year will decrease in the intervention group (IG) by 40% (or 15 days) compared to the control group (CG). One secondary outcome addresses the functional capacity. It is assumed that the functional capacity in the intervention group will be significantly ($p < 0.05$) higher than in the control group. In addition, patient and provider satisfaction with the intervention will be explored, including barriers, hurdles, and potentials. Direct and indirect costs are another outcome and will be compared between IG and CG.

For peer review only - http://bmjopen.bmj.com/site/about/guidelines.xhtml

Process evaluation

To evaluate the process quality, we will examine the assignment of participants to the standard and the complex program along with the distribution of the IG to low, medium and high intensity RP. In addition, we will consider usage of the modules (e.g., Medical Training Therapy, Psychological Support, Phone Consultation) as well as the app utilization. To examine the quality of the intervention, patients will receive additional questions about their satisfaction with individual MPT and RP modules within the survey. In order to evaluate experiences and satisfaction with the intervention, guided interviews will be conducted. These will be carried out with all physicians, psychologists and physiotherapists involved.

Health economic analysis

The health economic analysis will examine the costs, cost savings and cost-effectiveness of the intervention. Therefore, intervention costs, expenditures of health care utilization, indirect costs due to incapacity to work, and the patient's health-related quality of life will be assessed. For the calculation of indirect costs, the days of incapacity to work are multiplied by the average hourly wage in Germany, taking into account whether the individuals work full-time or part-time. The evaluation will be carried out based on claims data. Incremental cost-effectiveness will be analyzed by considering direct and indirect costs as well as the health-related quality of life of both groups, IG and CG.

Measurements

Various instruments will be used in the course of primary data collection, including standardized questionnaires (Table 1). Prior to the start of MPT, the intervention and control groups will receive a questionnaire to assess the status quo at the beginning of the intervention (T0). The questionnaire will be handed out directly at the Rehabilitation Center or sent by mail in individual cases for T0 and after the four-week MPT program (T1). It will comprise various items on socio-demographic and socio-economic parameters together with health-related behavior (exercise, diet, smoking etc.). Health-related Questionnaires will be the Heaviness of

For peer review only - http://bmjopen.bmj.com/site/about/guidelines.xhtml

Smoking Index (HSI) [22], the AUDIT alcohol consumption questionnaire, an effective brief screening test for problem drinking (AUDIT-C) [23] and the Pain Disability Index (PDI) [24]. In addition, the questionnaire will include items about how often and to what extent the study participants exercise [25], as well as questions about diet and nutrition [26]. In addition, standardized survey instruments will be included to assess functional capacity (Questionnaire for the diagnosis of functional disability caused by backache; FFbH-R) [27] and health-related quality of life (EQ-5D) [28]. A visual analog scale will be used to assess the patient's health-related quality of life from 0-100. IG and CG will receive further questionnaires twelve months post-intervention (T2). All questionnaires except for T0 will include questions regarding patient satisfaction and experience with the intervention. Two weeks after handing out or sending the questionnaire, patients who have not yet responded will receive a reminder to increase the response rate. A second reminder will be sent after an additional ten days. Reminders and the final questionnaires (T2) for the IG will be sent by the Rehabilitation Center, while those for the CG will be sent by the health insurance fund.

Secondary data will be included in the analyses based on claims data of the regional health insurance fund AOK Lower Saxony. These will include inpatient treatment, rehabilitation services, pharmaceuticals and remedies along with days of incapacity to work. Moreover, sociodemographic factors of influence (e.g. age, gender, level of education) together with data regarding health-related quality of life will be included in the analysis.

Table 1. Quantitative and qualitative data sources

Target group	Data source	Data type	Instrument	Parameter
**Patients**	Primary data	Quantitative	Questionnaire	Age, gender, sociodemography, satisfaction, functional capacity, health-related quality of life,

For peer review only - http://bmjopen.bmj.com/site/about/guidelines.xhtml

				impairment due to pain, health behavior, sports activity, nutrition, smoking, alcohol
**Medical Staff, Psychologists**	Primary data	Qualitative	Interview	Motivation, expectations, experiences, hurdles and barriers, suggestions for improvement and satisfaction with the intervention
**Patients**	Secondary data	Quantitative	Claims data	Days of incapacity to work, region, pharmaceuticals, therapeutic products, outpatient and inpatient care

For peer review only

Study population

The study population will comprise members of the regional health insurance fund AOK Lower Saxony, residing in the northwest of Lower Saxony (see Supplemental Figure S1). The specific inclusion and exclusion criteria are shown in table 2.

Table 2. Inclusion and exclusion criteria

**Inclusion criteria**	
Membership	AOK Lower Saxony
Region	Northwest of Lower Saxony (see Supplemental Figure S1)
Sick pay	Entitled

For peer review only - http://bmjopen.bmj.com/site/about/guidelines.xhtml

Incapacity to work	At least 21 days during the last six months
Diagnoses	Back pain (ICD: M40-M54, excluding M45, M46 and M49)
**Exclusion criteria**	
Age	≥ 62 years
Diagnoses	• Mental and behavioral disorders (ICD: F00-F99; six months before)<br>• Malignant neoplasms (ICD: C00-C97; twelve months before incapacity to work)<br>• Injuries (ICD: S00-S99; six months before incapacity to work)<br>• Chronic back pain (duration of the incapacity to work within the last six months is cumulatively longer than twelve weeks)

For peer review only

Participant recruitment

Each month, with the exception of December, two MPT courses with eight participants each can be held at the Rehabilitation Center Bad Zwischenahn. In December, a single course will be held. Over a recruitment period of 24 months, a maximum of 368 individuals could begin an MPT. Potentially eligible individuals will be identified and contacted through health insurance employees. If they are interested, they will be informed and invited to participate in the program. A diagnostic day will then be arranged at the Rehabilitation Center (see Supplemental Figure S1), where the medical criteria will be reviewed once again.

The risk factors of a possibly low willingness to participate or a high dropout rate of patients were counteracted in advance by the close involvement of the specialized employees of the AOK. For example, the inclusion and exclusion criteria were developed based on their experience. Nevertheless, the development of the case number and the dropout rate will be subject to constant monitoring so that actions can be taken and incentives implemented if necessary.

Sample Size calculation

The case number calculation was carried out for the primary outcome days of incapacity to work. Currently, the described patient group of AOK-insured persons has an average number of 37 days off work (SD: 58) per year. It is assumed that the average number of 37 days off

For peer review only - http://bmjopen.bmj.com/site/about/guidelines.xhtml

work per year will decrease in the intervention group (IG) by 40% (or 15 days) compared to the control group. (CG). This results in an effect size of d = 0.3409275. To test with a power of 80% (1 - ß = 0.8) and a significance level of 5% (α error = 0.05), a case number of 137 subjects per group is required for significant proof of the expected effect. The number of cases was calculated using the software G*Power (see Supplemental Figure S2).

Taking an intraclass correlation coefficient (ICC) of 0.01 and a design effect of 1.1 at an average cluster size of 15.3 (22*16+2*8) into account, the required number of cases is 293 subjects, i.e. 147 subjects per group [29–32]. Considering a drop-out of 10% for patients who drop out during the MPT, i.e., before randomization, results in a required total case number of 322. The available population covers the required case number (see Supplemental Figure S2).

Figure 3: CONSORT Flow Diagram.

Statistical analysis

Statistical analysis is performed on the basis of a defined analysis plan. If there are justified deviations from this plan, these will be documented and reported in subsequent publications. Compared to the original plan, the one-sided test with regard to hypothesis 4 was changed to a two-sided test, as this seems reasonable.

Qualitative analysis

The qualitative guided interviews will be documented by means of audio recordings. The recordings will be transcribed without personal information (e.g., name, place of residence). The evaluation of the guided interviews will be based on the qualitative content analysis according to Mayring and carried out with the help of the MAXQDA software [33].

Quantitative analysis

For peer review only - http://bmjopen.bmj.com/site/about/guidelines.xhtml

All patient-related data will be pseudonymized by assigning a unique identification number. A plausibility check of the data will be carried out. Initially, descriptive analysis of the data will be performed. Sample characteristics will be analyzed with regard to their distribution in the two groups by means of the Chi-square test for binary variables and Mann-Whitney-U-test for continuous variables.

Primary Outcome

The data will be analyzed by reference to the primary outcome (days of incapacity to work), which is carried out by intention-to-treat analysis. Patients are analyzed according to their original group assignment.

To test the primary hypothesis H_1 "The intervention reduces the number of days of incapacity to work", an analysis of covariances (ANCOVA) with cluster adjustment will be performed, using a significance level of 0.05. Covariables included into the model will be age, gender and comorbidities (e.g. depression). In addition, a hypothesis test (two-sided t-test with a significance level of 0.05) will be performed.

Secondary outcomes

Data for secondary outcome variables will be obtained via questionnaires and claims data. The Markov Chain Monte Carlo approach (MCMC) in SPSS will be used as imputation method to replace missing values [34–36]. To test the hypothesis H_2 "The intervention increases functional capacity" and H_4 "The intervention reduces direct and indirect costs", hypothesis tests (two-sided t-test with a significance level of 0.05) will be performed. The third hypothesis H_3 "The intervention is perceived positively by the participating patients" will be tested by conducting a pencil-paper questionnaire with the patients. The analyses are carried out using the software program SPSS.

Patient and Public Involvement

For peer review only - http://bmjopen.bmj.com/site/about/guidelines.xhtml

No patients were involved in the design and conduct of the study, choice of outcome measures and recruitment to the study. The results of the study will be provided to patients at their request.

ETHICS AND DISSEMINATION

Ethical and scientific standards in the currently valid version will be taken into account when conducting the study, especially the Memorandum on Good Scientific Practice of the German Research Foundation [37]. In particular, analysis of secondary data will be performed according to the Good Practice of Secondary Data Analysis guidelines, whereas the primary data collection will be conducted in accordance with the STROBE (Strengthening the Reporting of Observational Studies in Epidemiology) statement [38]. In addition, the methods of health economic evaluation in health services research and the Hannover Consensus will be applied [39]. Preliminary to the intervention, a concrete analysis plan is defined. Moreover, the questionnaires developed for the MPT and RP will be pre-tested and all data will be subjected to a validation process. In addition, participation in the intervention is voluntary for the insured, so there is no disadvantage if people do not want to participate. The applied treatments within the intervention and the potential health problems that may occur are known. Possible adverse events include for example pain intensification, pain relapse, muscle strain and other side effects that can occur with any physical activity and specific exercises. The practitioners are prepared and educated for possible adverse events. Experienced practitioners will immediately recognize when adverse events occur during the treatment. Moreover, in the case of an adverse event, they will react appropriately, report and document the adverse event in the electronic patient record. Appropriate action will be taken, such as discontinuation or modification of the treatment.

This study has received approval by the ethics committee of the Hannover Medical School (reference number 8548_BO_S_2019) and is registered at German Clinical Trials Register

For peer review only - http://bmjopen.bmj.com/site/about/guidelines.xhtml

DRKS00017654. Each participating patient will sign a contract for participation in the program, and we will obtain written informed consent. Furthermore, all study participants will be comprehensively informed about the use of the collected data and the purpose and procedure of the project. All transmitted data will be pseudonymized and is accessible only to project members. The delivered data will be stored on a secure and encrypted network drive. Access to the files is restricted to the project staff. No data will be passed on to third parties. Furthermore, the information obtained will be used exclusively for research purposes and no information will be passed on that enables conclusions about individual persons. All data collected and processed will be stored on a secure and encrypted hard disk after the end of the study and deleted ten years after (by 31 March 2033 at the latest).

The study results will be disseminated in national and international journals and conference presentations. The Innovation Fund (G-BA) will publish the study outcomes in a results report. Additionally, the health insurance fund AOK Lower Saxony will make the results available via its homepage and member journals.

For peer review only

Author contributions

KK and CK conceived, designed and obtained funding for the research. The first draft of this manuscript was produced by KK and JS. All authors have provided input to, reviewed, edited and approved the final version. Conceptualization: KK, CK, WR, GZ. Funding acquisition: CK, MP, NH. Methodology: KK, CK. Project administration: MP. Supervision: CK. Visualization: JS, KK. Writing - original draft: JS, KK. Writing - review & editing: JS, KK, CK, MP, WR, GZ, NH.

Funding statement

This project is funded by the Federal Joint Committee (G-BA), Innovation Fund, (funding code 01NVF18011). The funding company had no role in study design, data collection and analysis, decision to publish, or preparation of the manuscript.

Competing interests

The authors declare that they have no competing interest.

Abbreviations

AOK	Allgemeine Ortskrankenkasse (Regional Statutory Health Insurance Fund)
CG	Control group
IG	Intervention group
RP	Relapse prevention
LBP	Low Back Pain
MPT	Multimodal pain therapy

Appendix

Not available.

Acknowledgements

Not applicable.

For peer review only - http://bmjopen.bmj.com/site/about/guidelines.xhtml

REFERENCES

1 Casser H-R, Seddigh S, Rauschmann M. Acute Lumbar Back Pain. *Dtsch Arztebl Int* 2016;113(13):223–34.

2 Raspe H-H, ed. Rückenschmerzen. Berlin: Robert-Koch-Institut (RKI) 2012.

3 Neuhauser H, Ellert U, Ziese T. Chronische Rückenschmerzen in der Allgemeinbevölkerung in Deutschland 2002/2003: Prävalenz und besonders betroffene Bevölkerungsgruppen. *Gesundheitswesen* 2005;67(10):685–93.

4 Pengel LHM, Herbert RD, Maher CG, et al. Acute low back pain: systematic review of its prognosis. *BMJ* 2003;327(7410):323.

5 Mehling WE, Gopisetty V, Bartmess E, et al. The prognosis of acute low back pain in primary care in the United States: a 2-year prospective cohort study. *Spine (Phila Pa 1976)* 2012;37(8):678–84.

6 Hestbaek L, Leboeuf-Yde C, Manniche C. Low back pain: what is the long-term course? A review of studies of general patient populations. *Eur Spine J* 2003;12(2):149–65. doi:10.1007/s00586-002-0508-5 [published Online First: 28 January 2003].

7 Enthoven P, Skargren E, Oberg B. Clinical course in patients seeking primary care for back or neck pain: a prospective 5-year follow-up of outcome and health care consumption with subgroup analysis. *Spine (Phila Pa 1976)* 2004;29(21):2458–65.

8 Bundesärztekammer (BÄK), Kassenärztliche Bundesvereinigung (KBV), Arbeitsgemeinschaft der Wissenschaftlichen Medizinischen Fachgesellschaften (AWMF). Nationale VersorgungsLeitlinie Nicht-spezifischer Kreuzschmerz – Langfassung, 2. Auflage. Version 1 2017.

9 Wenig CM, Schmidt CO, Kohlmann T, et al. Costs of back pain in Germany. *Eur J Pain* 2009;13(3):280–86. doi:10.1016/j.ejpain.2008.04.005 [published Online First: 3 June 2008].

10 Schmidt CO, Raspe H, Pfingsten M, et al. Back pain in the German adult population: prevalence, severity, and sociodemographic correlates in a multiregional survey. *Spine (Phila Pa 1976)* 2007;32(18):2005–11.

11 Robert Koch-Institut. Gesundheit in Deutschland: RKI-Bib1 (Robert Koch-Institut) 2016.

12 Pfingsten M. Chronischer Rückenschmerz--Interdisziplinäre Diagnostik und Therapie. *Anästhesiol Intensivmed Notfallmed Schmerzther* 2009;44(1):40-5; quiz 46. doi:10.1055/s-0028-1128185 [published Online First: 29 December 2008].

For peer review only - http://bmjopen.bmj.com/site/about/guidelines.xhtml

13 Pfingsten M, Arnold B, Böger A, et al. Sektorenübergreifende interdisziplinäre multimodale Schmerztherapie Empfehlungen zu Struktur- und Prozessparametern der Ad-hoc-Kommission „Interdisziplinäre Multimodale Schmerztherapie“ der Deutschen Schmerzgesellschaft e. V. *Schmerz* 2019;33(3):191–203.

14 Arnold B, Brinkschmidt T, Casser H-R, et al. Multimodale Schmerztherapie: Konzepte und Indikation. *Schmerz* 2009;23(2):112–20.

15 Behrendt S, Kulas H, Marschall U, et al. Which Back Pain Patients Benefit From Multidisciplinary Pain Management? A Comparative Cost Analysis Using Claims Data of Statutory Health Insurance. *Gesundheitswesen* 2016;78(S 01):e120-e127. doi:10.1055/s-0042-110401 [published Online First: 21 July 2016].

16 Kamper SJ, Apeldoorn AT, Chiarotto A, et al. Multidisciplinary biopsychosocial rehabilitation for chronic low back pain: Cochrane systematic review and meta-analysis. *BMJ* 2015;350:h444. doi:10.1136/bmj.h444 [published Online First: 18 February 2015].

17 Bergström G, Jensen IB, Bodin L, et al. The impact of psychologically different patient groups on outcome after a vocational rehabilitation program for long-term spinal pain patients. *Pain* 2001;93(3):229–37.

18 Jensen IB, Busch H, Bodin L, et al. Cost effectiveness of two rehabilitation programmes for neck and back pain patients: A seven year follow-up. *Pain* 2009;142(3):202–08. doi:10.1016/j.pain.2008.12.015 [published Online First: 13 February 2009].

19 Michaelson P, Sjölander P, Johansson H. Factors predicting pain reduction in chronic back and neck pain after multimodal treatment. *Clin J Pain* 2004;20(6):447–54.

20 Nicholas MK, Asghari A, Sharpe L, et al. Cognitive exposure versus avoidance in patients with chronic pain: adherence matters. *Eur J Pain* 2014;18(3):424–37. doi:10.1002/j.1532-2149.2013.00383.x [published Online First: 13 August 2013].

21 Fuchs R, Goehner W, Seelig H. Long-term effects of a psychological group intervention on physical exercise and health: the MoVo concept. *J Phys Act Health* 2011;8(6):794–803.

22 Deutsche Version des Fagerström-Test for Nicotine Dependence (FTND-G) und des Heaviness of Smoking Index (HSI-G) 2003.

23 Bush K, Kivlahan DR, McDonell MB, et al. The AUDIT alcohol consumption questions (AUDIT-C): an effective brief screening test for problem drinking. Ambulatory Care Quality Improvement Project (ACQUIP). Alcohol Use Disorders Identification Test.

For peer review only - http://bmjopen.bmj.com/site/about/guidelines.xhtml

Archives of internal medicine 1998;158(16):1789–95. https://pubmed.ncbi.nlm.nih.gov/9738608/.

24 Dillmann U, Nilges P, Saile H, et al. Behinderungseinschätzung bei chronischen Schmerzpatienten. *Schmerz* 1994;8(2):100–10.

25 Dlugosch GE, Krieger W. Fragebogen zur Erfassung des Gesundheitsverhaltens: Swets Test Services 1995.

26 Keller S. Zur Validität des Transtheoretischen Modells eine Untersuchung zur Veränderung des Ernährungsverhaltens: Philipps-Universität Marburg 1998.

27 Kohlmann T, Raspe H. Der Funktionsfragebogen Hannover zur alltagsnahen Diagnostik der Funktionsbeeinträchtigung durch Rückenschmerzen (FFbH-R). *Rehabilitation* 1996;35(1):I–VIII.

28 J.-Matthias Graf v. d. Schulenburg, C. Claes, W. Greiner, A. Uber. Die deutsche Version des EuroQol-Fragebogens. *Zeitschrift für Gesundheitswissenschaften* 1998;6(1):3–20.

29 Eldridge SM, Ashby D, Kerry S. Sample size for cluster randomized trials: effect of coefficient of variation of cluster size and analysis method. *Int J Epidemiol* 2006;35(5):1292–300. doi:10.1093/ije/dyl129 [published Online First: 30 August 2006].

30 Dreyhaupt J, Mayer B, Kaluscha R, et al. Cluster-randomisierte Studien: Methodische und praktische Aspekte. *Rehabilitation* 2020;59(1):54–61. doi:10.1055/a-0801-5697 [published Online First: 23 January 2019].

31 Rutterford C, Copas A, Eldridge S. Methods for sample size determination in cluster randomized trials. *Int J Epidemiol* 2015;44(3):1051–67. doi:10.1093/ije/dyv113 [published Online First: 13 July 2015].

32 Lorenz E, Köpke S, Pfaff H, et al. Cluster-Randomized Studies. *Dtsch Arztebl Int* 2018;115(10):163–68.

33 Mayring P. Qualitative Inhaltsanalyse: Grundlagen und Techniken, 11th edn. Weinheim: Beltz 2010.

34 Li K-H. Imputation using markov chains. *Journal of Statistical Computation and Simulation* 1988;30(1):57–79.

35 Rubin BD. Multiple imputation for survey nonresponse 1987.

36 Mayer B. Fehlende Werte in klinischen Verlaufsstudien - der Umgang mit Studienabbrechern 2011.

For peer review only

For peer review only - http://bmjopen.bmj.com/site/about/guidelines.xhtml

37 Vorschläge zur Sicherung guter wissenschaftlicher Praxis: Empfehlungen der Kommission "Selbstkontrolle in der Wissenschaft" ; Denkschrift. Weinheim: Wiley-VCH 2013.

38 Graf von der Schulenburg J-M, Greiner W, Jost F, et al. German recommendations on health economic evaluation: third and updated version of the Hanover Consensus. *Value Health* 2008;11(4):539–44. doi:10.1111/j.1524-4733.2007.00301.x [published Online First: 11 January 2008].

39 Workgroup GPGI. Gute Praxis Gesundheitsinformation. *Z Evid Fortbild Qual Gesundhwes* 2016;110-111:85–92. doi:10.1016/j.zefq.2015.11.005 [published Online First: 13 January 2016].

For peer review only

For peer review only - http://bmjopen.bmj.com/site/about/guidelines.xhtml

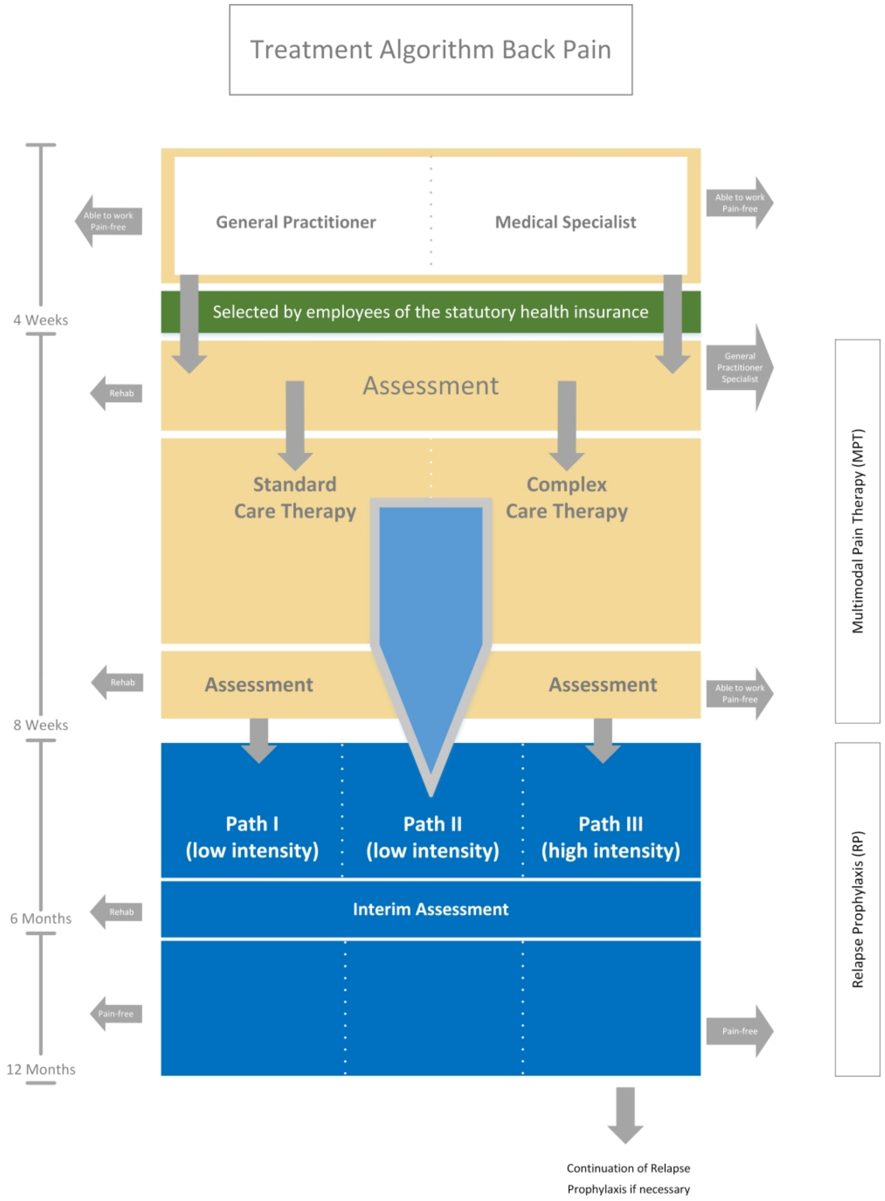

Treatment Algorithm

415x565mm (236 x 236 DPI)

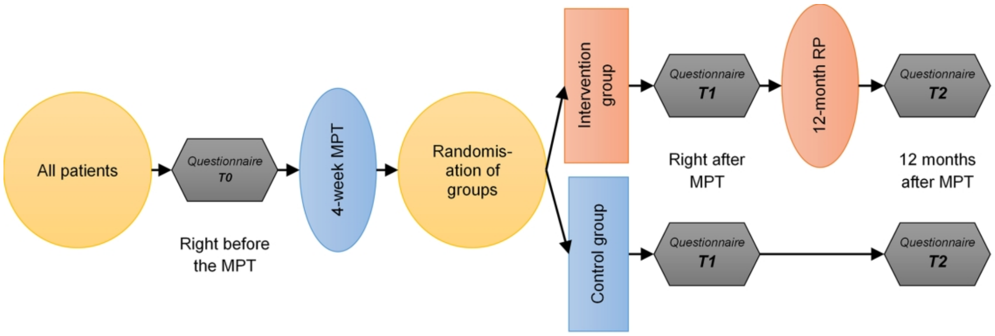

Study design

190x66mm (300 x 300 DPI)

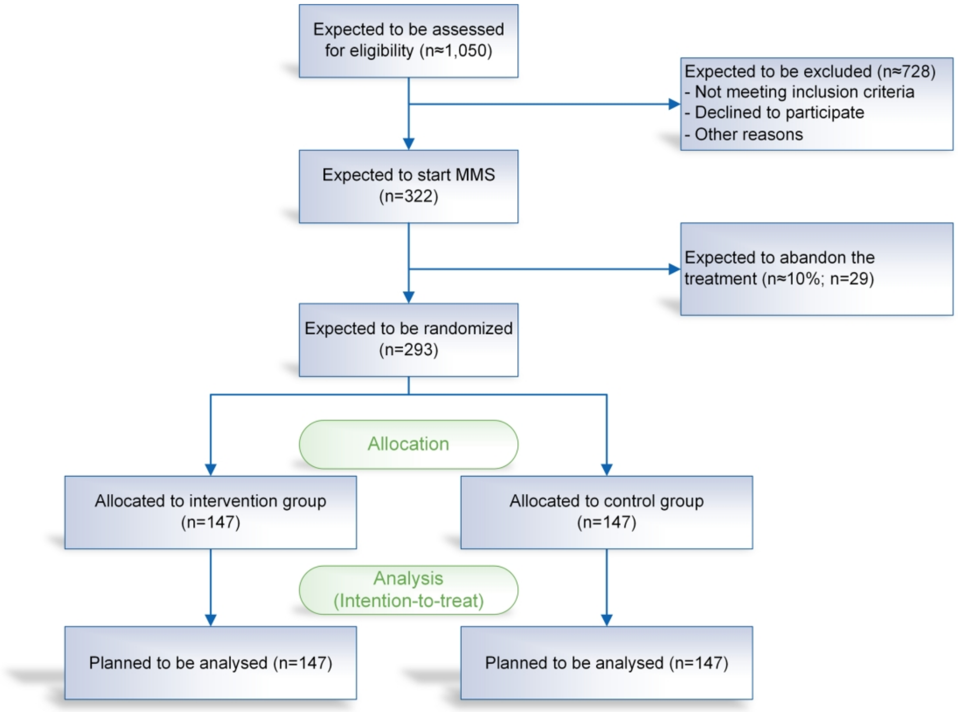

CONSORT Flow Diagram

205x147mm (300 x 300 DPI)

Germany study region lower saxony

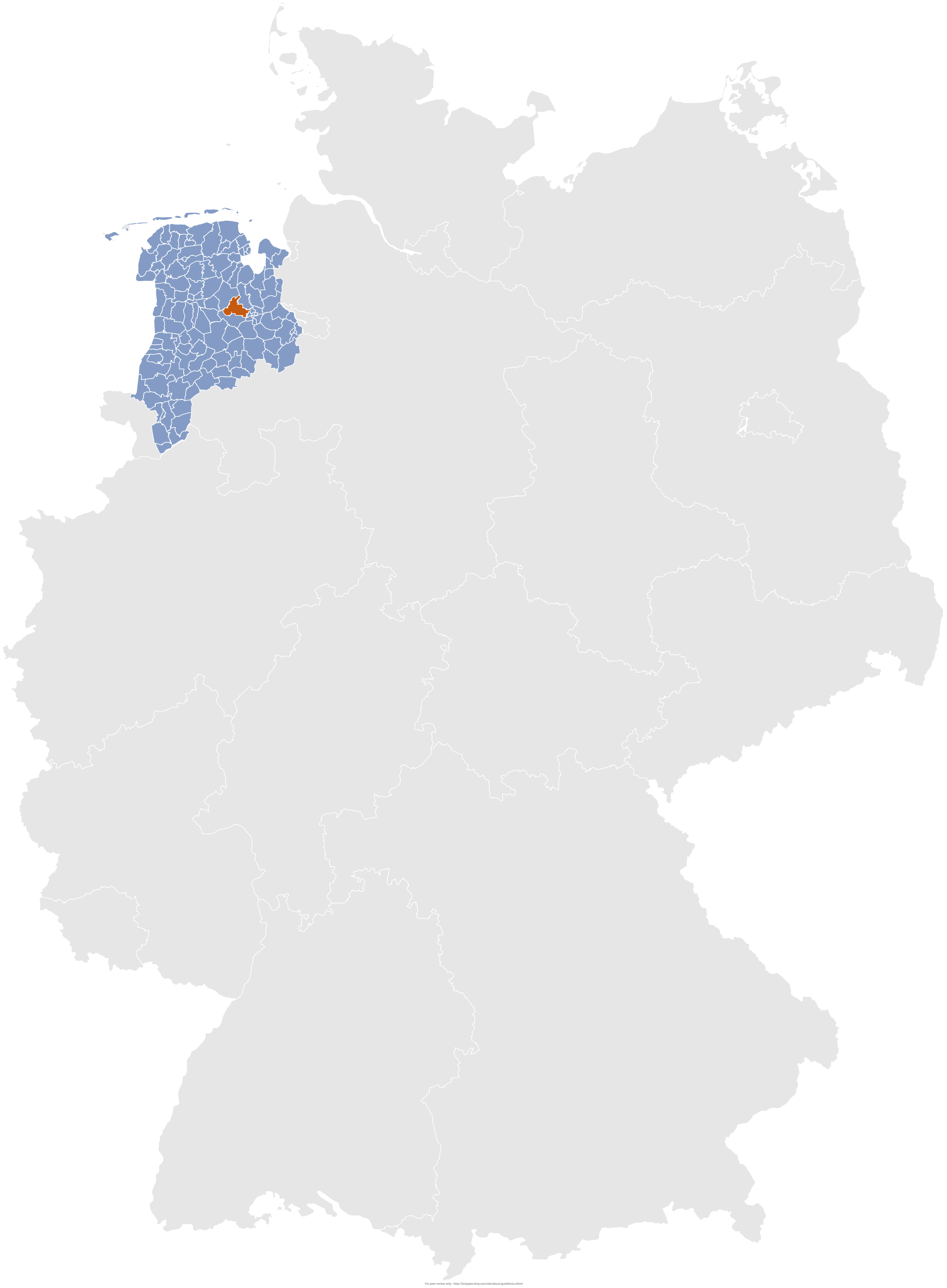

t tests – Means: Difference between two independent means (two groups)

**Analysis:**	A priori: Compute required sample size		
**Input:**	Tail(s)	=	Two
	Effect size d	=	0.3409275
	α err prob	=	0.05
	Power (1-β err prob)	=	0.80
	Allocation ratio N2/N1	=	1
**Output:**	Noncentrality parameter δ	=	2.8216771
	Critical t	=	1.9687238
	Df	=	272

70x37mm (300 x 300 DPI)
